# GlassPlane-SLAM: Glass-Plane-Aware RGB-D SLAM for Improved Localization in Glass Environments

**DOI:** 10.3390/s26217050

**Published:** 2026-11-07

**Authors:** Ziquan Wang, Zeyuan Chen, Souta Takahashi, Masahiko Mikawa, Makoto Fujisawa

**Affiliations:** 1 Graduate School of Comprehensive Human Sciences, University of Tsukuba, Tsukuba 305-8550, Ibaraki, Japan; chen.zeyuan.tkb_gu@u.tsukuba.ac.jp (Z.C.); s2621758@u.tsukuba.ac.jp (S.T.); 2 Institute of Library, Information and Media Science, University of Tsukuba, Tsukuba 305-8550, Ibaraki, Japan; mikawa@slis.tsukuba.ac.jp (M.M.); fujis@slis.tsukuba.ac.jp (M.F.)

**Keywords:** RGB-D SLAM, plane-based SLAM, glass surface depth estimation, RGB-D SLAM dataset

## Abstract

RGB-D simultaneous localization and mapping (SLAM) remains challenging in glass-rich environments with many glass walls and windows because depth measurements in glass regions are often missing, incorrect, or associated with background surfaces. Existing glass-aware methods mainly suppress unreliable observations in glass regions, leaving the glass surfaces themselves unused as landmarks in pose optimization despite their potential to provide additional geometric constraints. Meanwhile, conventional plane-based SLAM methods exploit planar structures to constrain camera motion, but they cannot reliably recover physical glass planes from raw RGB-D measurements because of glass transparency. To bridge this gap, we propose GlassPlane-SLAM, which recovers glass-plane geometry from noisy depth measurements of approximately coplanar surrounding structures and uses the recovered planes as shared landmarks to constrain camera motion across keyframes. A two-stage random sample consensus (RANSAC)-based procedure groups geometrically consistent glass blocks and refits unified planes from their aggregated measurements, reducing the influence of unreliable local observations on the estimated glass geometry. The resulting plane landmarks are jointly optimized with conventional point landmarks and camera poses in local bundle adjustment, while useful point correspondences are preserved. We further construct the UT-SRL Glass RGB-D Dataset to address the lack of RGB-D SLAM data covering long trajectories in glass environments. It contains 38 room-scale and large-scale sequences with pseudo-ground-truth trajectories estimated using FAST-LIVO2. /revBExperiments demonstrate improved localization accuracy on many glass-rich sequences, with particularly pronounced gains in large-scale environments. Qualitative mapping results further demonstrate improved geometric consistency in glass-rich environments.

## 1. Introduction

Simultaneous Localization and Mapping (SLAM) is a fundamental capability for autonomous mobile robots, enabling simultaneous pose estimation and map construction in unknown environments. It supports downstream tasks such as autonomous navigation and path planning. RGB-D cameras are particularly suitable for indoor robotics because they provide synchronized appearance and depth information, allowing visual observations to be directly associated with three-dimensional geometry. These advantages have made RGB-D SLAM a widely used approach for indoor robot localization and mapping [1,2,3,4].

**Figure 1:**
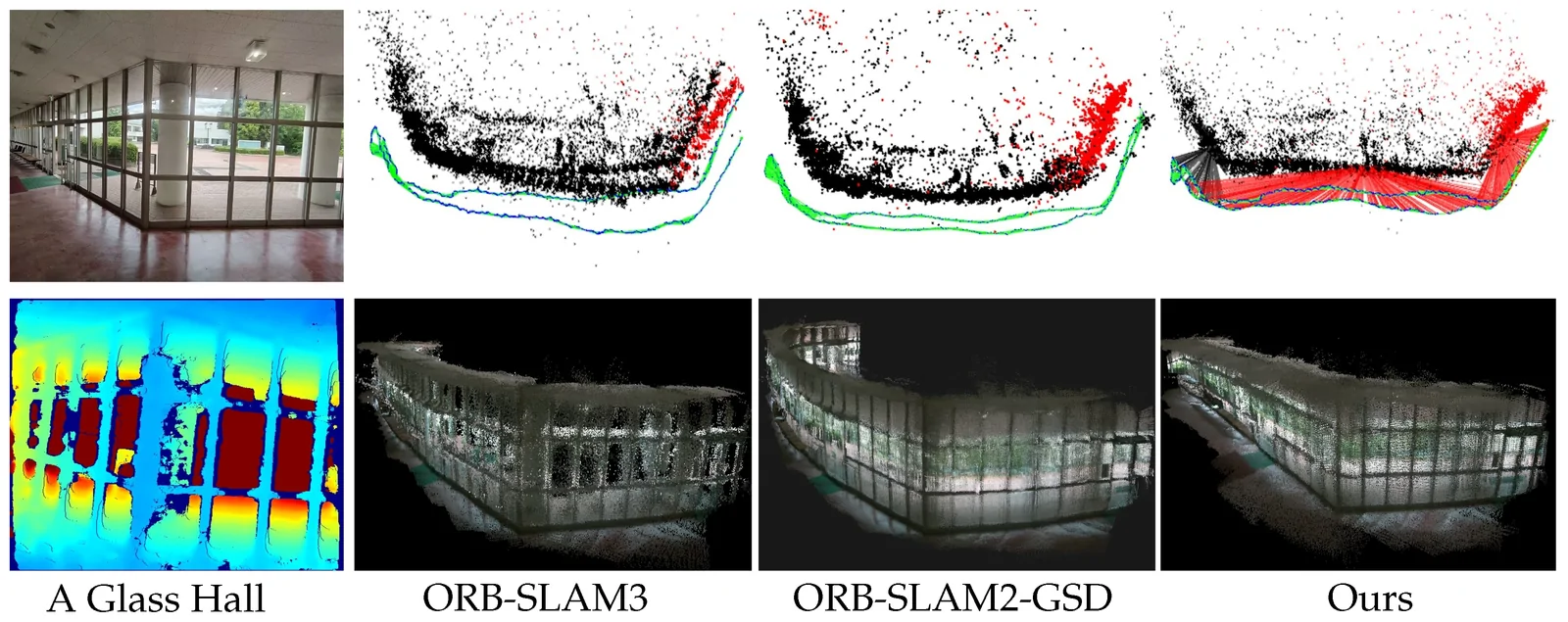
Comparison of SLAM mapping results in a glass scene. ORB-SLAM3 [4] and ORB-SLAM2-GSD [5] exhibit visible geometric distortion along the glass structures, reflecting accumulated pose inconsistency over the trajectory. With additional glass-plane constraints, our method maintains better alignment of the scene structure and improved local pose consistency.

However, environments containing glass structures, such as glass-walled lobbies and glass corridors, pose a fundamental challenge to RGB-D SLAM. Owing to the transparency and reflectivity of glass, depth measurements in glass regions may be missing, correspond to objects behind the glass or beyond the effective sensing range, or contain spurious values caused by reflections. Consequently, visual features detected in glass regions may be associated with depth measurements that do not represent the same physical surface. When such depth measurements are used to back-project visual features into 3D, the resulting map points can have incorrect positions and bias camera pose estimation. These pose errors can accumulate over the trajectory and become visible as geometric distortion in the reconstructed map, as illustrated in Figure 1.

Existing glass-aware RGB-D SLAM methods mainly focus on reducing pose errors caused by incorrect depth measurements in glass regions or reconstructing glass surfaces for map representation [5,6]. ORB-SLAM2-GSD [5], for example, filters out erroneous depth measurements associated with feature points in detected glass regions, excluding them from pose estimation. Although this strategy can reduce the influence of incorrect depth measurements, it may also reject valid measurements associated with static objects visible through the glass. When glass occupies a large portion of the image, excessive filtering may leave too few usable point constraints for camera pose estimation, potentially increasing trajectory drift. In addition, these approaches ignore the geometric information that glass surfaces themselves could provide for camera pose estimation.

Providing additional plane constraints in SLAM optimization has been shown to improve robustness when too few point features can be reliably tracked across frames [7,8,9,10,11,12]. This suggests a possible direction for addressing the reduced number of usable point constraints in glass-dominated environments. Existing plane-based SLAM methods incorporate planar landmarks fitted to groups of 3D points on surfaces such as walls, floors, and ceilings into pose optimization, providing spatially extended structural constraints in addition to conventional point observations. However, these methods cannot directly exploit glass surfaces because conventional plane extraction relies on sufficiently dense and geometrically consistent depth measurements from the observed surface. In glass regions, the measured depths generally do not represent the physical glass surface itself. Consequently, the actual glass plane may be missed or replaced by a plane fitted to background geometry. In glass-dominated environments, this limitation becomes particularly important because the remaining non-glass structures may provide insufficient geometric constraints for robust pose estimation.

To address these limitations, we propose GlassPlane-SLAM, which introduces glass surfaces themselves as planar landmarks for RGB-D SLAM optimization. The key idea is to use the geometry of glass surfaces as additional plane constraints on camera motion, rather than only suppressing unreliable observations in glass regions. The glass-plane landmarks are optimized together with conventional point landmarks, providing additional geometric constraints while useful point correspondences are preserved. This is particularly beneficial in glass-dominated environments, where reliable non-glass observations may be limited.

**Figure 2:**
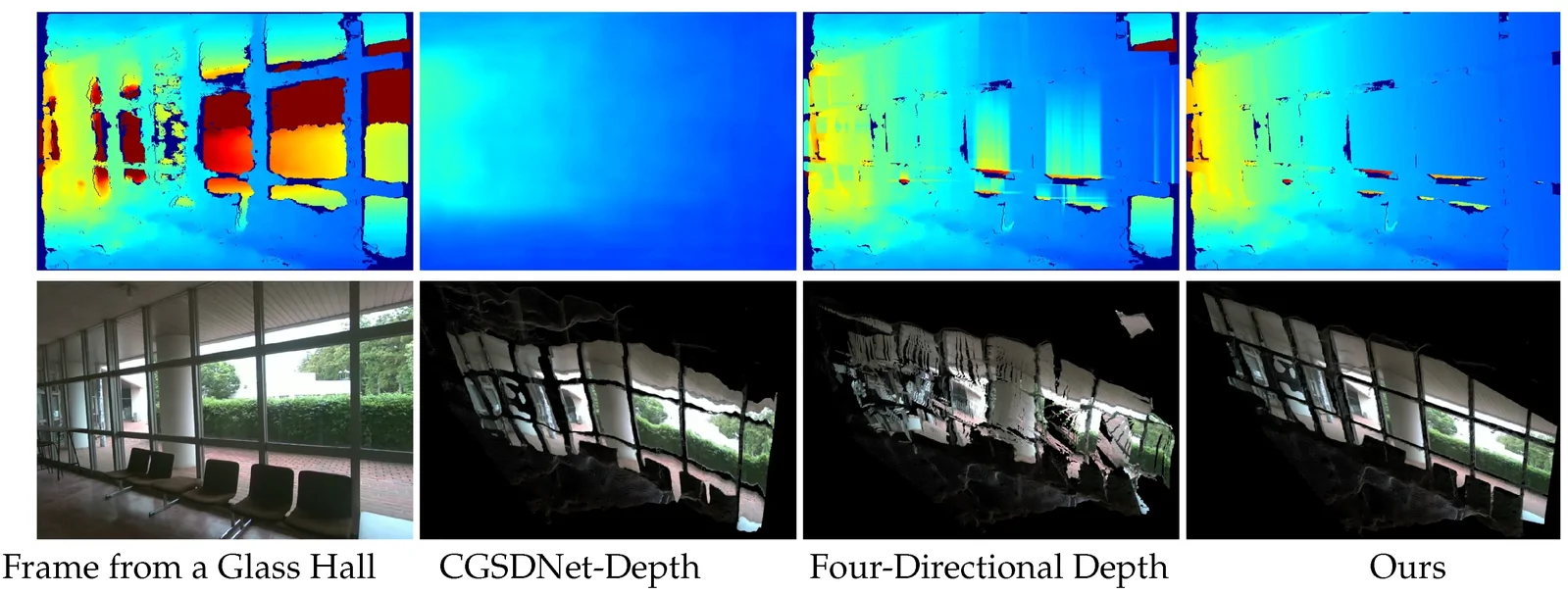
Comparison of glass-surface depth estimation results. The existing learning-based CGSDNet-Depth and image-processing-based Four-Directional Depth methods [5] may produce unstable or geometrically inconsistent estimates in complex glass scenes. Our method produces more consistent glass-surface geometry.

Implementing this idea requires reliable recovery of the geometry of the physical glass surface. Several learning-based methods have been developed to estimate glass-surface geometry from RGB observations [5,13,14]. However, since the glass geometry is inferred from learned visual models rather than directly obtained from sensor depth measurements, the resulting estimates may deviate from the true physical geometry, particularly when the test environment differs from the training data. Chen et al. [5] introduced a geometry-based method that estimates the depth of each glass pixel by taking a weighted combination of the nearest valid depth measurements found in the upward, downward, leftward, and rightward directions, referred to as the Four-Directional Depth method throughout this paper. Although it directly exploits metric sensor measurements, its pixel-wise estimation relies on a small number of local observations and is therefore sensitive to sensor noise, unreliable measurements, and occluded supporting structures. Examples of glass-depth estimation results are shown in Figure 2.

In GlassPlane-SLAM, we recover the glass-plane geometry from depth measurements of approximately coplanar structures surrounding each detected glass region. However, these surrounding depth measurements are themselves affected by sensor noise and occasional gross errors, making reliable glass-plane estimation challenging. A two-stage RANSAC [15] procedure is therefore used to obtain a stable plane estimate. The first stage fits a plane to measurements around each connected glass region (glass block) and rejects outliers, while the second stage groups blocks with compatible plane estimates and refits a unified plane from their aggregated measurements, reducing sensitivity to local sensor noise and block-level errors. The recovered glass planes are represented as planar landmarks and incorporated into local bundle adjustment together with conventional point landmarks. Repeated observations of the same physical glass surface are associated with a shared plane landmark, allowing the glass surface itself to provide geometric constraints on camera poses across local keyframes.

To evaluate localization in glass environments and address the lack of RGB-D SLAM datasets covering long trajectories in such environments, we further construct the UT-SRL Glass RGB-D Dataset, which contains 38 room-scale and large-scale sequences recorded in various scenes containing glass windows, walls, doors, and long glass corridors. We use trajectories estimated by FAST-LIVO2 [16], a LiDAR–visual–inertial odometry framework, as pseudo ground truth for localization evaluation.

The main contributions of this paper are summarized as follows:

- We explicitly incorporate recovered glass planes into RGB-D SLAM pose optimization, associating repeated observations with shared plane landmarks in local bundle adjustment to provide additional geometric constraints for localization.
- We propose a two-stage RANSAC-based method for estimating glass-surface depth from noisy sensor measurements. By aggregating measurements from geometrically consistent glass blocks and fitting a unified plane to each group, the method reduces the influence of local measurement errors.
- We construct the UT-SRL Glass RGB-D Dataset for localization evaluation in glass environments. The dataset contains 38 sequences, including 18 room-scale sequences and 20 large-scale sequences with extended spatial coverage.

Quantitative experiments show that the proposed glass-plane constraints improve localization accuracy across diverse glass environments, with particularly pronounced improvements in several large-scale sequences.

## 2. Related Work

### 2.1. Conventional RGB-D SLAM

Numerous RGB-D SLAM systems [17,18,19,20,21] have been developed for robust localization and mapping in diverse environments, with ORB-SLAM2 [3] and ORB-SLAM3 [4] serving as representative examples. In the RGB-D configurations of ORB-SLAM2 and ORB-SLAM3, depth measurements provided by the camera allow two-dimensional ORB feature points to be directly back-projected into three-dimensional map points, which are then jointly optimized with the camera poses. This framework enables accurate and robust pose estimation across a wide range of environments.

However, the accuracy of these systems relies on the assumption that each visual feature is associated with a valid depth measurement from the same physical surface. This assumption is frequently violated in glass regions, where visual features may arise from reflections or objects visible behind the glass, while their associated depths may be missing, erroneous, or correspond to different physical surfaces. Using such mismatched observations as three-dimensional map points introduces erroneous constraints into pose optimization, causing pose errors to accumulate and leading to unstable tracking and trajectory drift. This problem is particularly severe in glass-dominated environments, where reliable observations from non-glass regions are often too sparse to suppress the resulting errors.

To provide additional structural constraints when reliable point features are sparse or unevenly distributed, several plane-based SLAM methods have been proposed, including KDP-SLAM [7], the point–plane SLAM methods of Guo et al. [8,9], PlanarSLAM [10], VIP-SLAM [11], and Structure PLP-SLAM [12]. These methods incorporate planar landmarks into pose optimization, typically together with point or line features. Because planes extend over larger spatial regions than individual point features, they can provide broader and more persistent geometric constraints, enabling robust pose estimation even in low-texture environments with few reliable point observations.

However, existing plane-based SLAM methods typically extract planes from RGB-D point clouds and therefore rely on valid depth measurements. This reliance becomes problematic in glass regions, where depth measurements are often missing, erroneous, or correspond to background objects. As a result, glass surfaces cannot be reliably recovered as planar landmarks. Although these methods can still use floors, ceilings, and opaque walls, such structures may occupy only a limited portion of the field of view, particularly in glass-dominated environments. Large and spatially continuous structures, including glass walls, doors, and windows, therefore remain unused, leaving the SLAM system with insufficient geometric constraints for stable pose estimation.

Therefore, glass-aware RGB-D SLAM needs to address both erroneous observations and insufficient geometric constraints.

### 2.2. Glass-Aware RGB-D SLAM

Compared with conventional RGB-D SLAM, relatively few methods have been developed specifically for environments containing large glass surfaces. Zhao et al. [6] proposed an RGB-D SLAM framework based on ORB-SLAM2 that detects glass surfaces and incorporates the reconstructed glass planes into the map. Although this approach improves the representation of glass structures, the estimated planes are not incorporated into pose optimization, and the effect of glass observations on localization accuracy is not explicitly addressed. ORB-SLAM2-GSD [5] was subsequently proposed to improve SLAM accuracy in glass environments. It evaluates the reliability of ORB features in glass regions using glass masks and estimated glass depths and removes features that may adversely affect pose estimation. However, the improvement is achieved primarily through feature filtering, which may also discard useful visual correspondences because some observations in glass regions remain valid. When glass walls or windows dominate the field of view, removing features from these regions can further reduce the already limited number of usable map points. More importantly, this strategy does not exploit the glass surfaces themselves, which are often the most prominent and spatially continuous structures in glass-dominated scenes, as geometric landmarks for constraining camera motion.

### 2.3. Glass Surface Depth Estimation from RGB-D Observations

Using glass surfaces as geometric constraints in SLAM requires recovery of their physical geometry. Various sensing modalities have been investigated for glass-surface depth estimation, including LiDAR intensity, ultrasonic measurements, and acoustic–visual fusion [22,23,24,25,26,27]. This work focuses on RGB-D cameras because they provide pixel-aligned appearance and metric depth measurements that can be directly integrated into geometric estimation and SLAM.

Nevertheless, glass remains difficult to measure using RGB-D cameras. The emitted infrared light may pass through the glass or be specularly reflected away from the receiver, resulting in missing depth measurements, erroneous values, or depths corresponding to objects behind the glass. To recover the actual depths of glass surfaces from these unreliable observations, several methods based on RGB or RGB-D data have been proposed.

Several learning-based methods estimate the depths of transparent objects from RGB or RGB-D observations [28,29,30,31]. However, these methods mainly target small transparent objects for robotic manipulation and grasping, rather than large-scale architectural glass structures such as glass walls, doors, and windows.

To address architectural glass scenes, several learning-based methods, including GW-Depth [32], CGSDNet-Depth [5], LayeredDepth [13], and MonoGlass3D [14], have been proposed to estimate glass-surface depth from RGB or RGB-D observations. Although these methods can infer dense glass depths when direct measurements are unavailable, the resulting estimates may deviate from the true physical geometry, particularly when the test environment differs from the training data. Such deviations may introduce inaccurate geometric constraints when the estimated surfaces are used in SLAM pose optimization.

In addition to learning-based approaches, Chen et al. [5] proposed the Four-Directional Depth method, which estimates glass-surface depth from valid measurements surrounding each glass region. For each glass pixel, the method searches for valid depths in the upward, downward, leftward, and rightward directions and estimates its depth using a weighted combination of these measurements. This method directly exploits metric depth measurements from the RGB-D sensor. However, reflections from surrounding structures and occasional depth-sensing failures may produce grossly incorrect measurements, while ordinary RGB-D sensor noise causes even valid measurements to fluctuate around their true values. Since each glass pixel is estimated independently from only a few local observations, these errors can directly propagate to the estimated depths, resulting in incomplete, discontinuous, or geometrically inconsistent glass surfaces. Moreover, although architectural glass structures are commonly planar, this pixel-wise formulation does not explicitly enforce a unified geometric model over the entire glass region.

Overall, existing RGB- and RGB-D-based methods can recover glass-surface depths to some extent, but errors in the recovered geometry can limit their reliability as constraints for SLAM pose optimization.

### 2.4. Summary and Research Gaps

Glass-aware SLAM methods based on point filtering primarily treat observations in glass regions as unreliable and reduce their influence on pose estimation, leaving the structural information of glass surfaces themselves unused for pose optimization. Conventional plane-based SLAM can exploit planar structures as geometric constraints, but cannot reliably recover physical glass planes from raw RGB-D measurements. Recovering glass planes from surrounding depth measurements is also challenging because these measurements are affected by sensor noise and occasional gross errors. These limitations motivate our proposed system, GlassPlane-SLAM. We first group glass blocks with compatible plane estimates and refit unified planes from their aggregated surrounding depth measurements, reducing the influence of local measurement errors. The recovered planes are then explicitly introduced as shared landmarks across keyframes and jointly optimized with conventional point landmarks and camera poses in local bundle adjustment, allowing the glass surfaces themselves to provide additional constraints on camera motion.

## 3. Glass Surface Depth Estimation

### 3.1. Challenges and Glass-Plane Estimation Strategy

Since direct depth measurements on glass surfaces are often unreliable, we use depth measurements from surrounding structural regions, such as window frames, door frames, and adjacent walls, to estimate the corresponding glass-plane geometry.

A key challenge is that RGB-D measurements of window frame regions can also be unreliable, especially when the frames are made of metal or other reflective materials. Specular reflections may cause clearly incorrect depth measurements, producing **outliers** that are far from the true surface position. In contrast, **random sensor noise** usually appears as small depth fluctuations around the true value, even when the same point is observed repeatedly. These fluctuations tend to become larger as the sensing distance increases.

We use RANSAC-based plane fitting to reduce the influence of these measurement errors. The plane model is estimated from the dominant geometric structure of the surrounding measurements, allowing gross outliers and local depth fluctuations to have less influence on the final estimate. The fitting is performed in two stages. The first stage operates on each individual glass-block region to reject gross outliers and obtain a block-level plane estimate. After blocks belonging to the same physical plane are grouped, the second stage estimates a unified plane from their aggregated measurements, further reducing the influence of local sensor noise and block-level estimation errors.

Our method relies on two assumptions. First, the target glass surfaces are approximately planar. Second, the majority of valid depth measurements around a detected glass region are assumed to originate from surrounding structural elements that are approximately coplanar with the glass surface, while foreground occlusions account for only a limited portion of the sampled measurements.

### 3.2. Two-Stage RANSAC Glass-Plane Estimation

**Figure 3:**
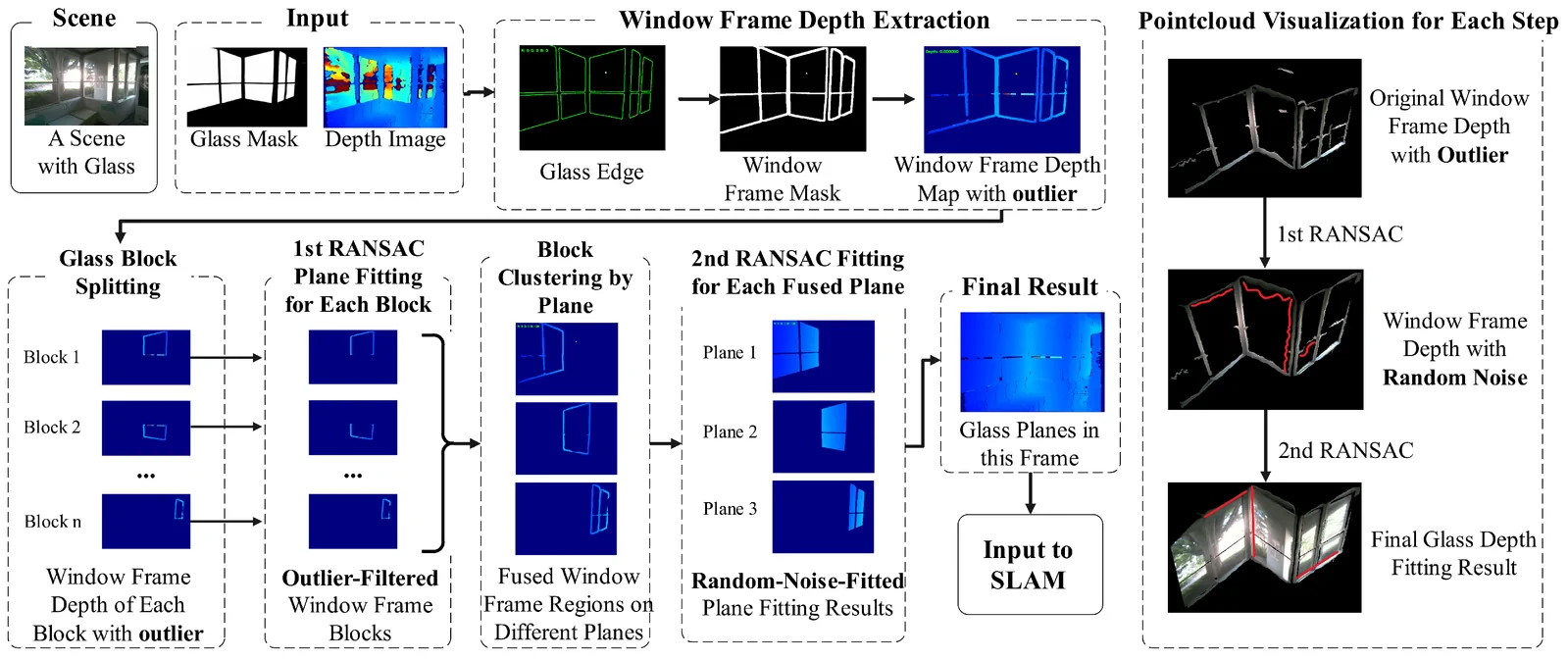
(**Left**): The proposed framework for glass-surface depth estimation. The input scene contains three glass planes. Given a glass mask and a depth image, the system applies two-stage RANSAC filtering and outputs the three distinct glass planes contained in the image, which are then used as inputs to the SLAM system. (**Right**): Visualization of the filtering process. The first RANSAC stage removes gross outliers from individual glass-block observations. After geometrically consistent blocks are grouped, the second stage refits a unified plane using the aggregated measurements, reducing the influence of local measurement noise and block-level estimation errors. The red wavy and straight lines illustrate the change from locally fluctuating measurements to a more consistent fitted surface.

We use a glass scene containing three glass surfaces lying on different planes as an example to illustrate the proposed method, as shown in Figure 3. The method takes the current depth image and a glass mask generated by the CGSDNet glass-segmentation network [33] as input. First, the connected components of the glass mask are extracted, with each component treated as an individual glass block and as the basic unit for plane estimation. For each glass block, valid depth measurements are extracted from the surrounding structural regions, such as window frames, around its boundary.

A two-stage RANSAC procedure is then applied to estimate the glass planes. In the first stage, the depth measurements associated with each glass block are back-projected into 3D to construct a local point cloud. RANSAC-based plane fitting is independently applied to the point cloud of each block, and the plane model with the largest inlier set is selected as the corresponding block-level plane. Consequently, each glass block is assigned an individual plane estimate, while measurements inconsistent with the estimated plane are rejected as outliers, reducing the influence of unreliable RGB-D measurements.

The resulting block-level planes are subsequently clustered according to their geometric similarity. Specifically, normal agreement and consistency of the predicted depths at both glass-region centers are used to determine whether two blocks belong to the same physical plane. By grouping geometrically consistent block-level planes into larger plane clusters, the proposed method can simultaneously estimate multiple glass planes within a single frame.

In the second RANSAC stage, the 3D measurements retained after first-stage filtering are aggregated across all glass blocks in each cluster, and a unified plane is fitted to the combined point cloud. This cluster-level fitting reduces local estimation errors caused by sensor noise and produces a plane estimate that is consistent across all glass blocks within the cluster.

Finally, the estimated plane parameters and the corresponding glass-surface depths are provided to the SLAM system as the glass-plane observations of the current frame.

## 4. Glass-Plane-Based Visual SLAM

### 4.1. Proposed SLAM Framework

To improve the robustness and accuracy of SLAM in glass environments, we develop a glass-plane-based visual SLAM framework that incorporates estimated glass planes as additional geometric landmarks. Once extracted from each keyframe, local glass-plane observations are associated with plane landmarks maintained in the global map. These associations establish plane observation constraints between keyframes and plane landmarks. These constraints are incorporated into local bundle adjustment together with conventional point-feature constraints to jointly refine camera poses, point landmarks, and plane landmarks.

### 4.2. Plane Representation and Transformation

An infinite plane π in 3D space can be represented as $\pi ={\left(n^{T},d\right)}^{T}$, satisfying $n^{T}p+d=0$. Here, $n={\left(a,b,c\right)}^{T}$ is the plane normal. Because multiplying all plane parameters by a nonzero scalar does not change the represented plane, the normal vector is normalized throughout this paper such that ∥n∥=1. Under this normalization, *d* represents the signed distance from the coordinate origin to the plane.

The RANSAC-based plane fitting described in Section 3 produces local plane observations in the camera coordinate system. Following the terminology of keypoints, each local plane observation is referred to as a *keyplane*. A *plane landmark*, in contrast, represents a physical plane maintained in the map and is expressed in the world coordinate system.

Before a keyplane can be associated with a plane landmark, it is transformed from the camera coordinate system to the world coordinate system as

(1) $${\tilde{\pi }}_{w}=T_{wc}^{-T}\pi_{c}=\left[\begin{array}{c} R_{cw}^{T}n_{c}\\ n_{c}^{T}t_{cw}+d_{c} \end{array}\right],$$

where $T_{wc}$ denotes the rigid-body transformation from the camera coordinate system to the world coordinate system, and $\left(n_{c},d_{c}\right)$ and $\left(n_{w},d_{w}\right)$ denote the plane parameters in the camera and world coordinate systems, respectively. Throughout this paper, $T_{ab}$ denotes the rigid-body transformation from coordinate frame *b* to coordinate frame *a*; accordingly, $T_{wc}=T_{cw}^{-1}$.

### 4.3. Plane Matching

To associate a newly observed keyplane with an existing plane landmark, the keyplane is first transformed into the world coordinate system using the plane transformation defined above. Because (n,d) and (−n,−d) represent the same physical plane, the transformed keyplane ${\tilde{\pi }}_{w}={\left({\tilde{n}}_{w}^{T},{\tilde{d}}_{w}\right)}^{T}$ is sign-aligned with the candidate plane landmark $\pi_{w}={\left(n_{w}^{T},d_{w}\right)}^{T}$ before matching. Specifically, if ${\tilde{n}}_{w}^{T}n_{w}<0$, we set $\left({\tilde{n}}_{w},{\tilde{d}}_{w}\right)\leftarrow \left(-{\tilde{n}}_{w},-{\tilde{d}}_{w}\right)$.

To assess spatial consistency, the centroid of each keyplane is computed from the 3D points back-projected from its estimated glass depths and transformed into the world coordinate system. For a plane landmark, the centroid is initialized from a keyplane observation and updated as map points are associated with it. These map points are selected from the observing keyframes if their perpendicular distances to the plane are less than 0.3 m. The two planes are considered a match if

(2) $${\tilde{n}}_{w}^{T}n_{w}>\tau_{\cos},\;d_{\mathrm{match}}<\tau_{d}.$$

Here, $d_{\mathrm{match}}$ is the maximum of the distances from each centroid to the other plane. When only the keyplane centroid is available, its distance to the landmark plane is used; when the keyplane centroid is unavailable, the offset difference $|{\tilde{d}}_{w}-d_{w}|$ is used instead. The thresholds are set to $\tau_{\cos}=0.9$ and $\tau_{d}=0.5$ m to accommodate moderate variations caused by sensor noise and pose-estimation errors.

If no existing plane landmark satisfies both criteria, the keyplane is retained as a candidate and a new landmark is created after confirmation through consecutive keyframe observations. Otherwise, it is associated with the matched landmark and a plane observation edge is added to the optimization graph. Multiple keyplanes observed in different keyframes can therefore be associated with the same plane landmark, providing additional constraints for the joint optimization.

### 4.4. Minimal Plane Parameterization

A plane has three degrees of freedom, whereas the representation (n,d) contains four parameters subject to the unit-normal constraint ∥n∥=1. Directly optimizing this over-parameterized representation requires explicitly handling the unit-normal constraint and complicates the parameter update. We therefore adopt the commonly used minimal parameterization based on azimuth, elevation, and signed distance [8,9,10]. Specifically, the normal vector n is represented by two angles (ψ,ϕ), where ψ denotes the azimuth angle of the projection of n onto the xy-plane, measured from the *x*-axis, and ϕ denotes its elevation angle with respect to the xy-plane. Let

(3) $$R_{n}=\underset{R_{z}\left(\psi \right)}{\underset{︸}{\left[\begin{array}{ccc} \cos\psi & -\sin\psi & 0\\ \sin\psi & \cos\psi & 0\\ 0 & 0 & 1 \end{array}\right]}}\underset{R_{y}\left(-\phi \right)}{\underset{︸}{\left[\begin{array}{ccc} \cos\phi & 0 & -\sin\phi \\ 0 & 1 & 0\\ \sin\phi & 0 & \cos\phi \end{array}\right]}},$$

Then the normal vector can be represented as

$$n=R_{n}\left[\begin{array}{c} 1\\ 0\\ 0 \end{array}\right]=\left[\begin{array}{c} \cos\;\psi \cos\;\phi \\ \sin\;\psi \cos\;\phi \\ \sin\phi \end{array}\right].$$

This equation transforms the unit vector along the *x*-axis by first applying a rotation of −ϕ around the *y*-axis and then a rotation of ψ around the *z*-axis. Here, $R_{z}\left(\psi \right)$ and $R_{y}\left(-\phi \right)$ denote the corresponding Euler-angle rotation matrices, and $R_{n}$ denotes the overall transformation.

Conversely, the minimal representation $\xi_{\pi }$ of a plane π can be obtained as

(4) $$\xi_{\pi }=\left[\begin{array}{c} \Theta \left(n\right)\\ d \end{array}\right]=\left[\begin{array}{c} \psi \\ \phi \\ d \end{array}\right]=\left[\begin{array}{c} atan2(b,a)\\ atan2\left(c,\sqrt{a^{2}+b^{2}}\right)\\ d \end{array}\right],$$

where Θ(n) denotes the mapping from a three-dimensional normal vector to its two-dimensional minimal angular representation. The angular parameterization becomes singular when $a^{2}+b^{2}=0$, corresponding to the normal vector approaching ${\left[0,0,\pm 1\right]}^{T}$, for which the azimuth angle is undefined. In practice, prior to residual computation and variable update, the normal vector is transformed into a local coordinate system in which it lies near ${\left[1,0,0\right]}^{T}$. The optimization is therefore performed away from these singular configurations.

### 4.5. Plane Error Calculation and Update on Manifolds

The plane parameters have a product-manifold structure: the unit normal vector n lies on the spherical manifold $S^{2}$, whereas the signed distance *d* lies in the Euclidean space R. Therefore, directly computing the plane error as $\pi_{1}-\pi_{2}$ treats both components as Euclidean variables, violates the manifold structure of n, and may lead to discontinuities or numerical instability during optimization.

Following the plane error formulation adopted in Structure PLP-SLAM [12], an inter-plane difference operator ⊖ is defined such that the discrepancy between two normal vectors is represented by the differences in their azimuth and elevation angles. Similarly, an update operator ⊕ is defined to apply small perturbations δψ and δϕ to the normal vector, together with a distance increment δd, during plane optimization. For two planes $\pi_{1}$ and $\pi_{2}$, their difference is computed as

(5) $$\pi_{1}⊖\pi_{2}=\left[\begin{array}{c} \Theta \left(R_{n_{2}}^{T}\cdot n_{1}\right)-\underset{0}{\underset{︸}{\Theta \left(R_{n_{2}}^{T}\cdot n_{2}\right)}}\\ d_{1}-d_{2} \end{array}\right],$$

where $R_{n_{2}}^{T}$ is the inverse of the Euler-angle rotation matrix introduced above. It rotates the normal vector $n_{2}$ back to (1,0,0). Applying the same inverse transformation to $n_{1}$ places it near (1,0,0), after which the angular difference can be computed. As indicated in the equation, the angular representation of the transformed $n_{2}$ becomes a zero vector. For the distance term *d*, we directly compute the difference. The resulting three-dimensional error jointly represents the local angular discrepancy of the plane normals and the difference in signed distance.

Similarly, during plane optimization, when a plane π is updated using a small increment δψ,δϕ,δd, we use the following update rule:

(6) $$\xi_{\pi }⊕\left[\begin{array}{c} \delta \psi \\ \delta \phi \\ \delta d \end{array}\right]=\left[\begin{array}{c} \Theta \left(R_{n}\cdot R_{\delta \psi ,\delta \phi }\cdot \left[\begin{array}{c} 1\\ 0\\ 0 \end{array}\right]\right)\\ d+\delta d \end{array}\right].$$

### 4.6. Point–Glass-Plane Bundle Adjustment

**Figure 4:**
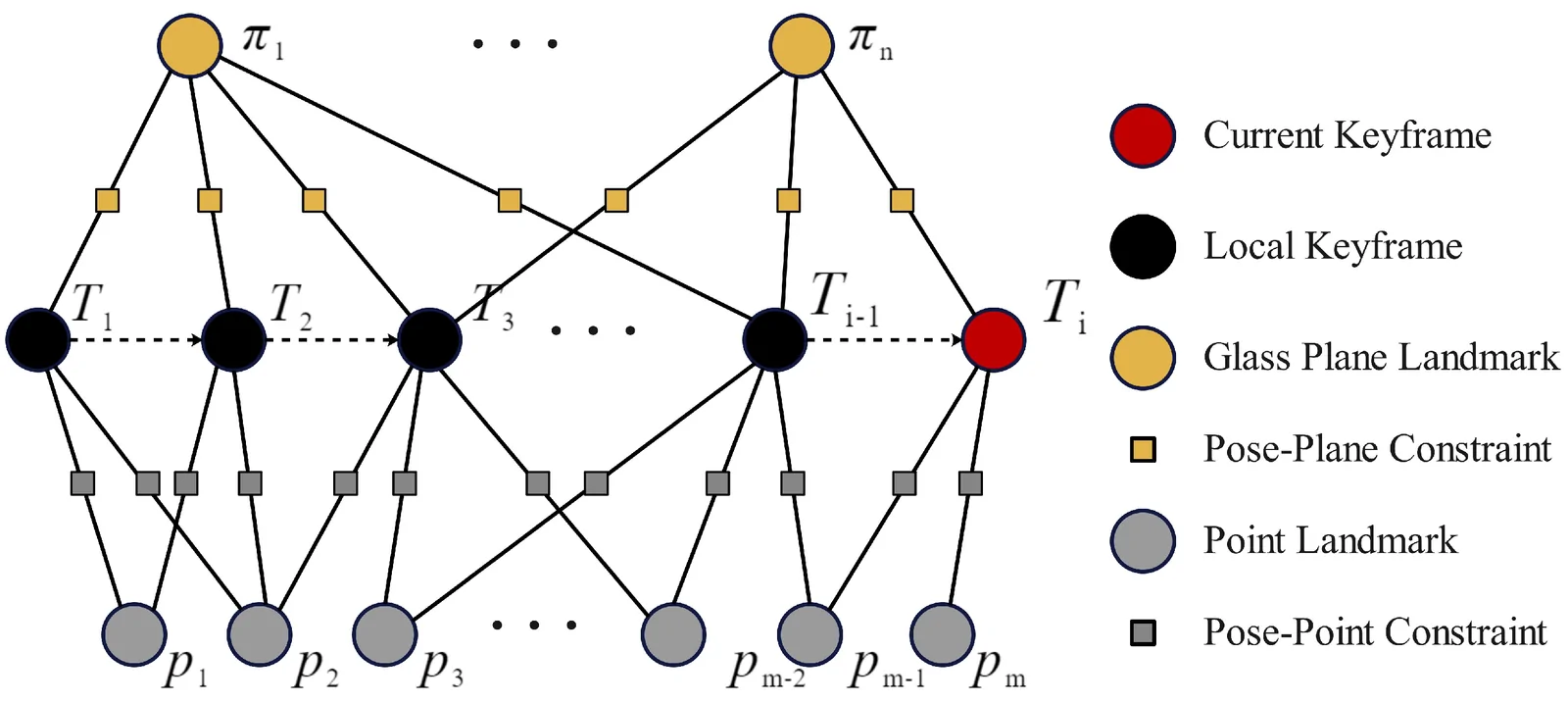
Illustration of the proposed local graph optimizer. Camera poses are jointly optimized with point and glass-plane landmarks through pose–point and pose–plane constraints. Multiple observations of the same glass surface can be associated with a common plane landmark, providing additional geometric constraints across local keyframes.

We represent detected glass surfaces as plane landmarks and incorporate them into local bundle adjustment. As illustrated in Figure 4, the proposed local graph optimizer jointly optimizes the poses of local keyframes, including the current keyframe, and the point and glass-plane landmarks observed within the local map. Pose–point edges provide conventional reprojection constraints, while pose–plane edges introduce additional geometric constraints from the detected glass surfaces. Since the same glass plane can be observed in multiple keyframes, multiple plane observations can be associated with a common plane landmark and jointly constrain the local camera poses and glass-plane geometry. The resulting point–glass-plane bundle adjustment is formulated as

(7) $$\underset{T^{*},p^{*},\xi_{\pi }^{*}}{\arg\min}\left(\sum_{i=1}^{K}\sum_{p\in K_{i}}H_{p}\left(e_{p}\right)+\sum_{i=1}^{K}\sum_{\pi \in K_{i}}H_{\pi }\left(e_{\pi }\right)\right),$$

where $H_{p}\left(\cdot \right)$ and $H_{\pi }\left(\cdot \right)$ denote the Huber robust kernel functions [34] for point and plane observations, respectively. The inner summation aggregates the observations in each keyframe, while the outer summation aggregates all keyframes whose observations are included in the local optimization. Boundary keyframes observing local point landmarks are held fixed to anchor the optimization.

For point observations, the reprojection error is defined as

(8) $$e_{p}={∥u_{\mathrm{obs}}-\rho \left(T_{cw}P_{w}\right)∥}_{\Omega_{p}}^{2},$$

where the weighted squared norm is defined as ${∥r∥}_{\Omega }^{2}=r^{T}\Omega r$. Here, $\Omega_{p}$ denotes the information matrix of the point observation, $u_{\mathrm{obs}}$ denotes the observed image position, $P_{w}$ denotes the corresponding point landmark in the world coordinate system, and ρ(·) denotes the camera projection model. For a 3D point $P_{c}={\left[X,Y,Z\right]}^{T}$ in the camera coordinate system, the projection model is

$$\rho \left(\left[\begin{array}{c} X\\ Y\\ Z \end{array}\right]\right)=\left[\begin{array}{c} f_{x}X/Z+c_{x}\\ f_{y}Y/Z+c_{y} \end{array}\right],$$

where $f_{x}$ and $f_{y}$ are the focal lengths and $\left(c_{x},c_{y}\right)$ denotes the principal point.

For a plane observation $\pi_{c}$ associated with a plane landmark $\pi_{w}$, the plane residual is defined as

(9) $$e_{\pi }={∥\pi_{w}⊖\left(T_{wc}^{-T}\pi_{c}\right)∥}_{\Omega_{\pi }}^{2},$$

where $T_{wc}^{-T}\pi_{c}$ transforms the observed plane from the camera coordinate system to the world coordinate system, and ⊖ denotes the plane difference operator defined above, in which Θ(·) is used to compute the normal-direction discrepancy in the two-dimensional minimal angular space.

Plane observations are sparse relative to point observations, and their residuals differ in numerical scale. Their contribution to the joint objective is controlled by the plane information matrix

(10) $$\Omega_{\pi }=\lambda I_{3},$$

where λ is empirically set to $10^{3}$ to account for the imbalance in constraint numbers, as the optimization graph contains substantially fewer plane edges than point edges. This value is kept fixed throughout the main experiments. Its scene-dependent effect is examined in the sensitivity analysis in Section 6.8; it is not assumed to be optimal for every scene. A Huber robust kernel is applied to suppress erroneous plane observations. The base threshold is empirically set to $\sqrt{7.815}$; after applying the weighting λ, the threshold is set to

$$\delta_{\pi }=\sqrt{7.815\lambda }.$$

The threshold is scaled by $\sqrt{\lambda }$ to maintain the same robust-kernel cutoff under the weighted plane error.

## 5. UT-SRL Glass RGB-D Dataset

### 5.1. Dataset Overview

Numerous RGB-D SLAM datasets, such as TUM RGB-D [35], have been developed for evaluating localization and mapping, while datasets specifically targeting glass-rich environments remain scarce. Chen et al. [5] collected glass-scene RGB-D data using a camera mounted on a robotic arm, whose motion is restricted to a range of approximately 0.5 m. This limits the evaluation of accumulated localization error over longer trajectories, motivating a glass-scene SLAM dataset covering more diverse environments and larger spatial scales.

We therefore construct the UT-SRL Glass RGB-D Dataset, containing 38 sequences collected in diverse glass-rich environments. The dataset includes both room-scale scenes with local glass structures and large-scale scenes extending beyond 10 m, such as glass corridors, dining halls, long glass walls, and building entrances with glass doors. Each sequence provides synchronized RGB and depth images.

The 38 sequences comprise 18 room-scale and 20 large-scale recordings from 17 acquisition scene groups. Recording durations range from 15.1 to 342.0 s, and the available FAST-LIVO2 reference paths range from 3.59 to 130.98 m. Table 1 summarizes each sequence’s duration, reference-path length, predicted glass pixel ratio, physical glass-plane count, loop-detection indicator, and lighting conditions.

**Figure 5:**
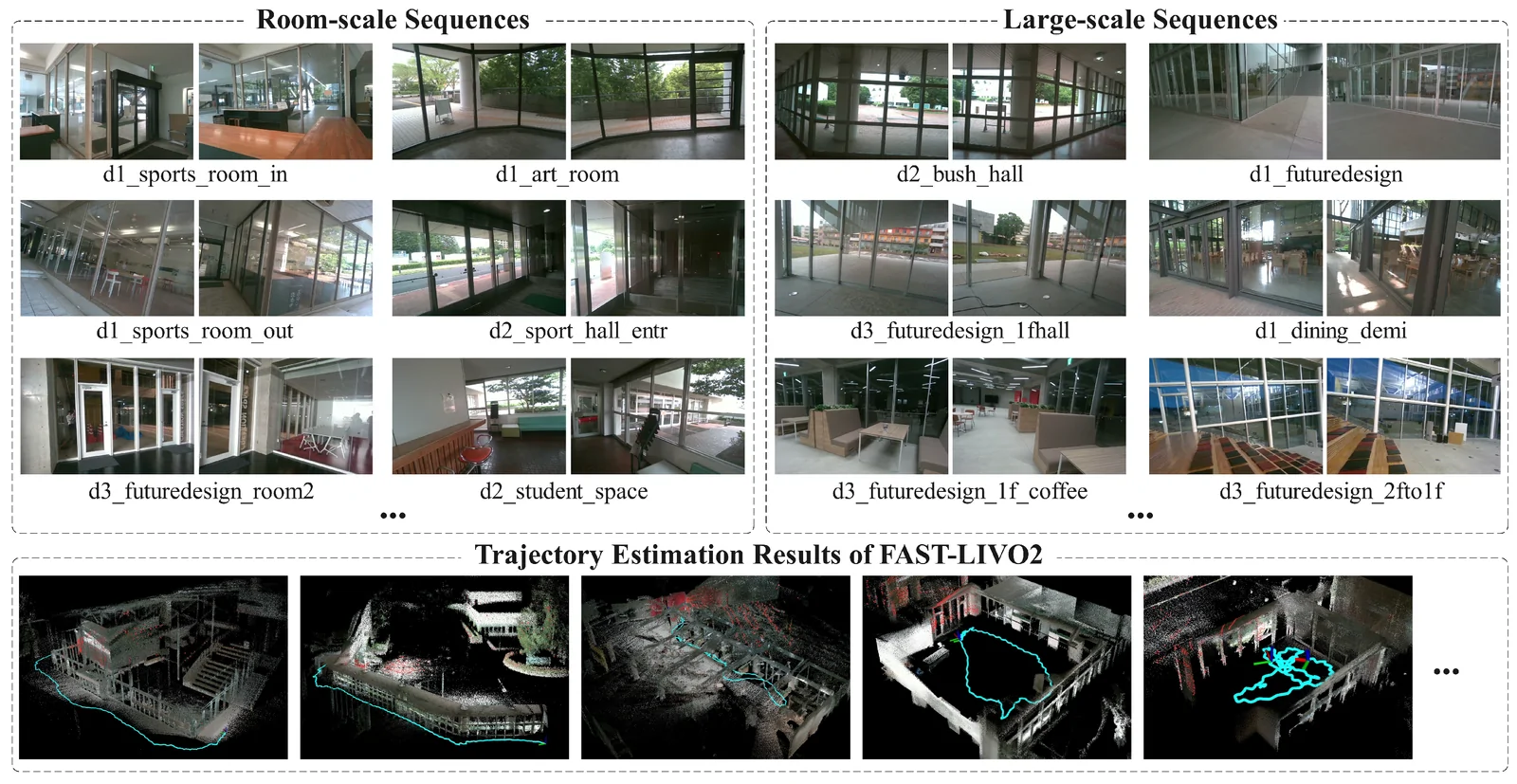
(**Top**): Overview of the proposed dataset, which contains 38 room-scale and large-scale sequences captured in diverse glass environments. (**Bottom**): Example FAST-LIVO2 trajectory (blue) and corresponding mapping result. The estimated trajectories are included in the dataset and used as pseudo ground truth for evaluation.

To obtain reference camera trajectories across diverse and large-scale glass-rich environments, we use LiDAR–visual–inertial odometry to generate pseudo-ground-truth trajectories, taking advantage of the accurate and robust geometric measurements provided by LiDAR compared with vision-only estimation. Specifically, synchronized LiDAR data are recorded together with the RGB-D sequences, and FAST-LIVO2 [16] is used to estimate the reference trajectories. Representative examples from the dataset, together with the corresponding reference trajectories and FAST-LIVO2 maps, are shown in Figure 5.

### 5.2. Data Acquisition

**Figure 6:**
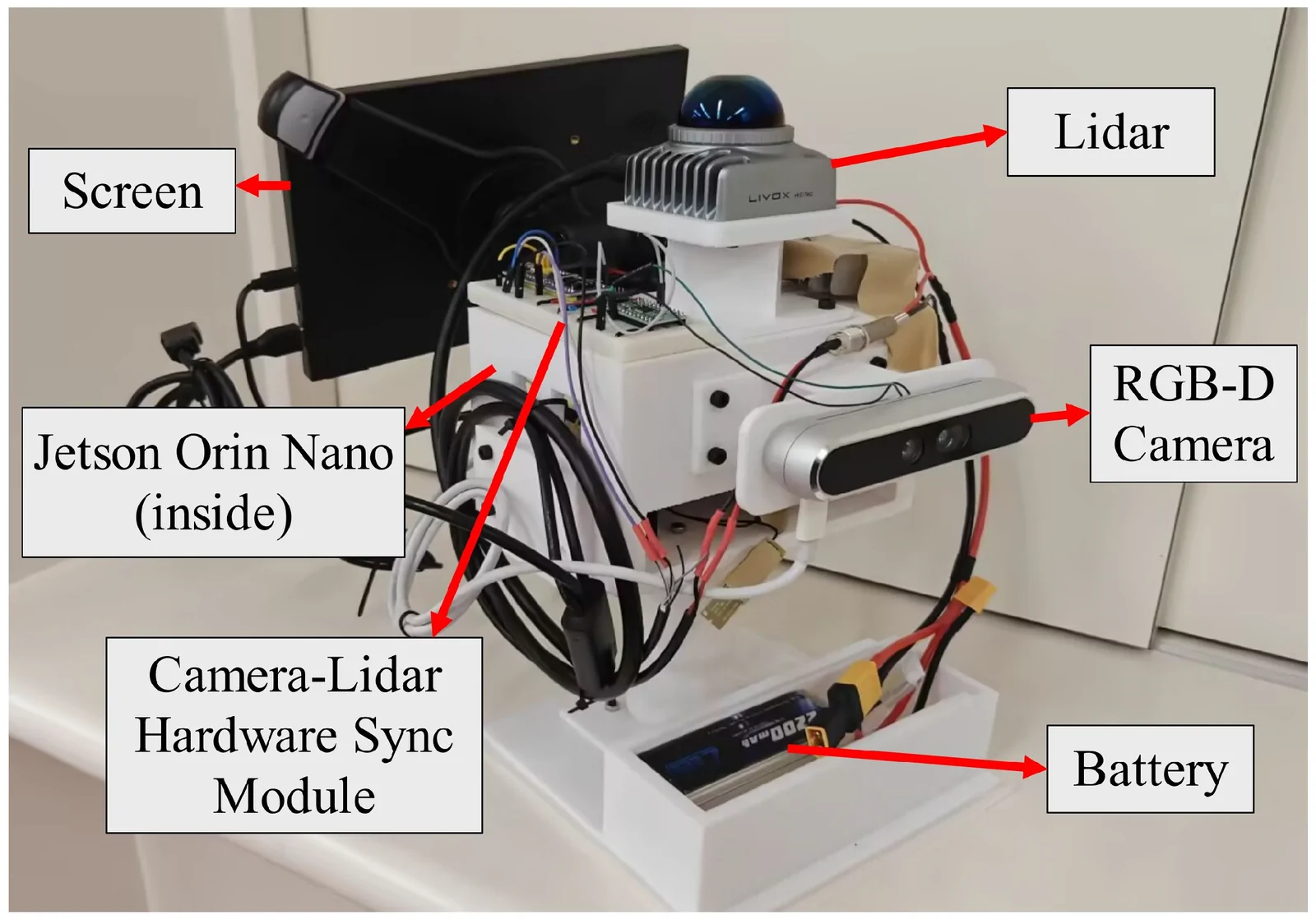
Data acquisition platform for synchronized RGB-D and LiDAR data collection. The system consists of a Livox MID-360 LiDAR, a RealSense D455f depth camera, a camera-LiDAR hardware synchronization module, and a Jetson Orin Nano.

Our data acquisition platform is shown in Figure 6. The system consists of a Livox MID-360 LiDAR (Livox Technology Company Limited, Hong Kong, China), a RealSense D455f depth camera (RealSense, Inc., Cupertino, CA, USA), and a Jetson Orin Nano (NVIDIA Corporation, Santa Clara, CA, USA). Both the LiDAR and the camera are connected to the Jetson Orin Nano for data collection. The camera captures images at 30 Hz, while the LiDAR records data at 10 Hz.

To achieve accurate temporal synchronization, we design a hardware synchronization scheme between the RealSense camera and the MID-360. As external trigger signals do not provide sufficiently stable control over RGB imaging on the RealSense camera, we configure the RealSense camera in master mode so that it actively outputs a 30 Hz synchronization signal. This signal is then converted by an STM32 into a 1 Hz PPS signal and the corresponding GPS time information, which are transmitted to the MID-360 to synchronize the LiDAR clock with the camera. The resulting synchronization provides a common time base for the RGB-D and LiDAR measurements.

After each sequence is recorded as a ROS 2 bag using ROS 2 Humble Hawksbill (Open Robotics, San Jose, CA, USA), FAST-LIVO2 [16] is applied to estimate the trajectory of the IMU integrated into the LiDAR. The estimated trajectory is then transformed into the camera coordinate system using the calibrated LiDAR–camera extrinsic parameters and included in the dataset as the pseudo-ground-truth camera trajectory.

### 5.3. Reference Trajectory Reliability and Discussion

The reliability of the reference trajectories is assessed qualitatively from the geometric consistency of the LiDAR reconstructions. As shown in Figure 5, the maps reconstructed by FAST-LIVO2 remain spatially continuous over the recorded trajectories, while major rigid structures such as walls and window frames are consistently reconstructed without apparent large-scale misalignment or deformation. These results indicate that the estimated trajectories provide a reasonably stable reference for the environments considered in this study. A complementary quantitative assessment using fixed mounting bases and independently measured distances is presented in Section 6.6.

Because the reference trajectories are estimated by a SLAM system, residual estimation errors may still be present. We therefore treat them as pseudo ground truth and interpret small differences in absolute trajectory error conservatively. In our experiments on several challenging sequences, substantial trajectory deviations are accompanied by clearly observable deformation or misalignment in the reconstructed scene. In such cases, the reference trajectories remain useful for identifying and comparing significant localization drift among the evaluated methods.

## 6. Experiments

### 6.1. Experimental Overview and Setup

We evaluate the glass-surface depth estimation module qualitatively and quantitatively, using reference planes constructed from depth measurements of opaque boards for the quantitative evaluation, and then assess the reliability of the FAST-LIVO2 reference trajectories using fixed mounting bases and independently measured distances. The complete SLAM system is evaluated on the UT-SRL Glass RGB-D Dataset through localization, mapping, ablation, loop-closing, and dense-reconstruction experiments. We also measure the runtime of the glass-surface depth estimation module. All experiments are conducted on a computer equipped with an AMD Ryzen 9 9900X CPU (Advanced Micro Devices, Inc., Santa Clara, CA, USA), an NVIDIA RTX 5060 Ti GPU (NVIDIA Corporation, Santa Clara, CA, USA), and 32 GB of memory.

### 6.2. Implementation Details

Glass-surface depth estimation is performed offline in the current implementation. For each detected glass region, candidate depths are extracted from a 10-pixel neighborhood outside the boundary, and an 8-pixel support band is used to assess surrounding structural depth. A valid side must contain at least 30 depth measurements covering at least 25% of its length.

Both RANSAC stages perform up to 80 sampling attempts, use three points per hypothesis, require at least 10 valid input points, and use a point-to-plane inlier threshold of 0.08 m. After the first stage, block-level planes are connected when the absolute inner product of their normals is at least 0.85 and their predicted depth difference is at most 0.20 m at both glass-region centers; connected observations are grouped by union–find. Groups covering less than 10% of the detected glass area are rejected. For each retained group, the filtered measurements are merged, the second RANSAC stage refits the plane, and glass depth is recovered by ray–plane intersection. Except for the dedicated weight-sensitivity experiment, the parameters of our method are kept fixed across all evaluated sequences without sequence-specific tuning.

### 6.3. Qualitative Glass Surface Depth Comparison and Glass Segmentation Analysis

**Qualitative comparison of glass surface depth estimation.**

**Figure 7:**
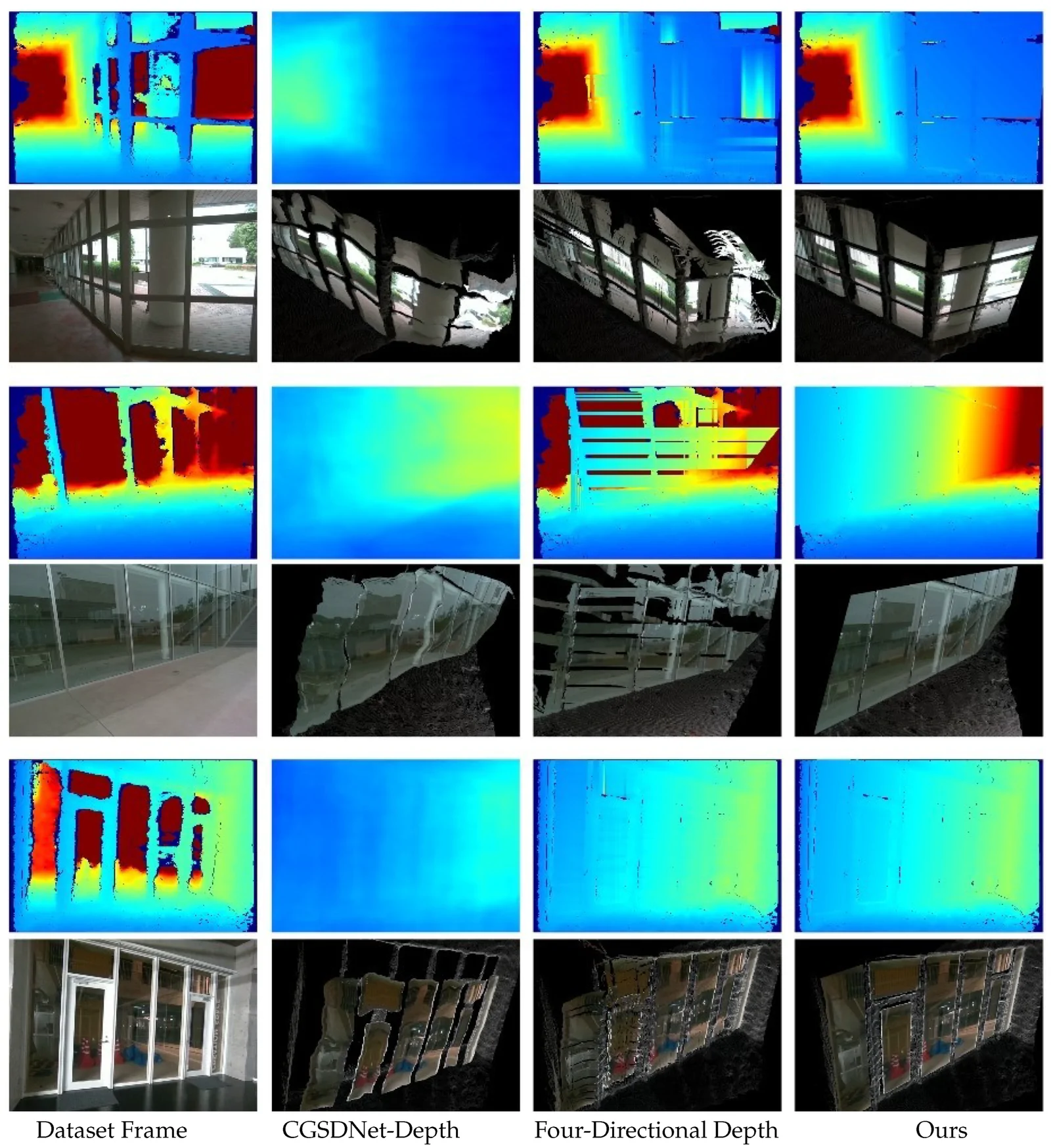
Visual comparison of glass depth estimation results among our method, the learning-based CGSDNet-Depth, and the Four-Directional Depth method in three representative scenes [5]. For each example, the first row shows the estimated depth map of the current frame, while the second row shows the corresponding visualized point cloud.

Figure 7 shows the glass-depth estimation results and corresponding point-cloud visualizations obtained using CGSDNet-Depth [5], Four-Directional Depth [5], and our method.

CGSDNet-Depth produces nonplanar glass surfaces or surfaces that are misaligned with the surrounding window frames in several examples. A likely cause is that its prediction depends on learned appearance and depth priors, so performance can degrade when the test scenes differ from the training distribution. In addition, the predicted depths are not explicitly constrained to form a single plane over an architectural glass region. To reduce these sensitivities, our method estimates the glass plane directly from metric depth measurements in the surrounding structures of the current scene and imposes a unified planar model, making the result less dependent on scene appearance and more geometrically constrained.

By comparison, Four-Directional Depth estimates each glass pixel from only a few nearby measurements. Noise or an incorrect depth selected along one search direction can therefore propagate directly to that pixel, while neighboring pixels may be influenced by different measurements, producing local discontinuities or an uneven surface. To reduce this sensitivity to local measurements, our method aggregates a larger set of surrounding measurements, rejects inconsistent samples with RANSAC, and assigns a common plane to geometrically consistent glass regions. This reduces the influence of individual noisy measurements and explains the smoother planar geometry observed in Figure 7.

Despite these improvements, the proposed method still depends on sufficient valid structural measurements around the glass region. Severe foreground occlusion can leave too few measurements supporting the true glass plane and degrade the RANSAC estimate. Future work will investigate the use of multi-frame geometric information during glass-plane estimation for such cases.

**Glass segmentation accuracy and its implications for depth estimation.** Glass segmentation errors can affect glass surface depth estimation because our method uses the predicted masks to identify window-frame regions and extract their depth measurements. A comprehensive evaluation of segmentation accuracy is difficult because glass-mask ground truth is unavailable for the full dataset. To quantify segmentation accuracy on a subset, we manually annotate three images from each of the 17 acquisition scenes, giving 51 images. Table 2 reports glass-class intersection over union (IoU), precision, and recall after pooling pixels across all annotated images. The saved 8-bit CGSDNet predictions are binarized at >120, consistent with contour and window-frame extraction.

**Figure 8:**
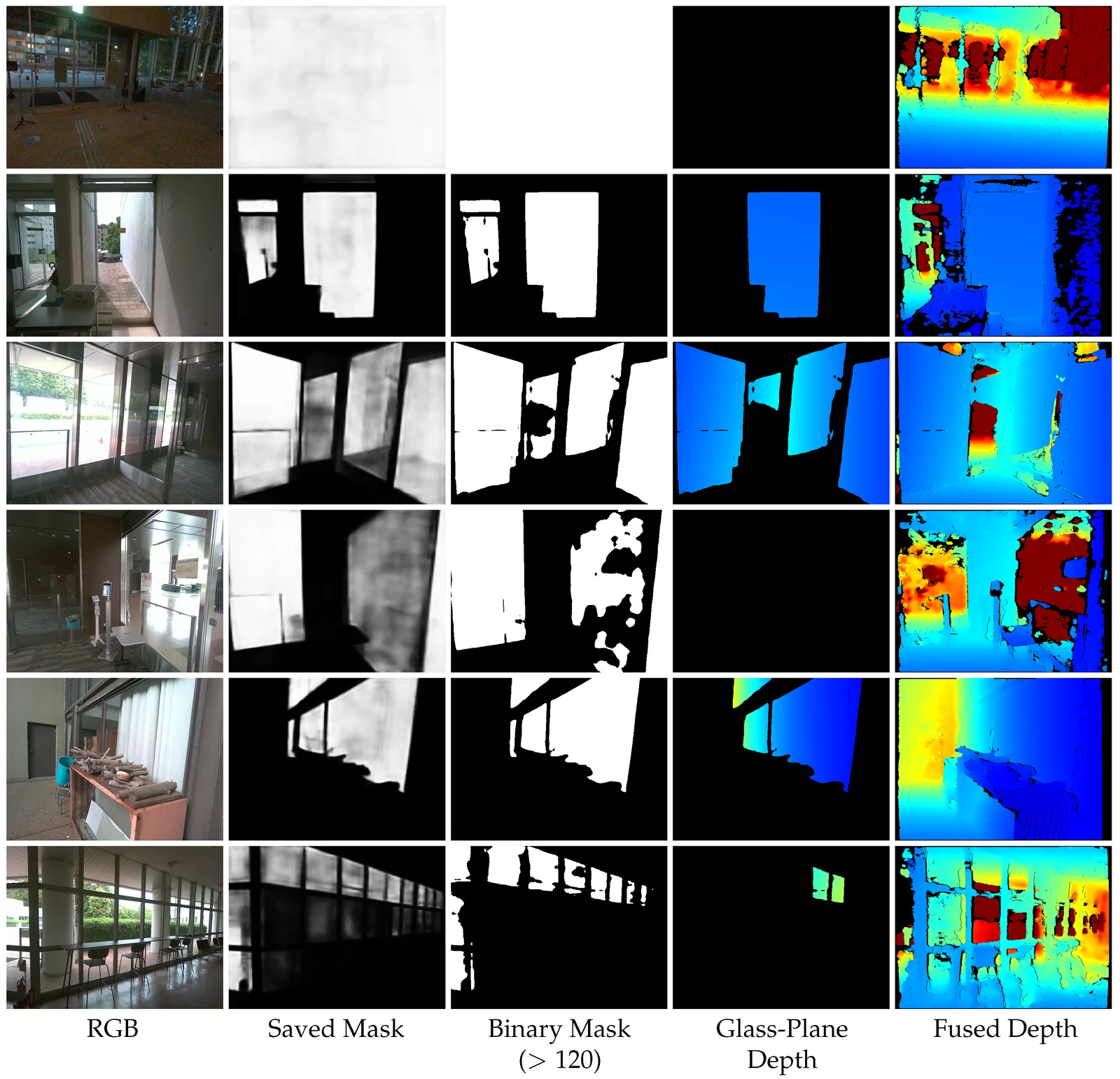
Representative glass-segmentation failures and the resulting depth estimates. Columns show the RGB image, saved grayscale prediction, binary mask at >120, estimated glass-plane depth, and fused depth. Plane candidates with insufficient window-frame depth support are rejected, reducing coverage. Black indicates no depth output.

Figure 8 illustrates representative mask failures and their effects on depth estimation. Reliable glass-depth estimation requires stable window-frame depths, while extracting these measurements depends on an accurate glass mask. Inaccurate masks may therefore leave plane candidates without sufficient depth support. Such candidates are filtered out to reduce the risk of introducing unreliable plane constraints into SLAM, at the cost of lower glass-depth coverage. Some examples yield no glass-plane output, while others retain only candidates with sufficient support.

### 6.4. Quantitative Evaluation of Glass Surface Depth Estimation

**Figure 9:**
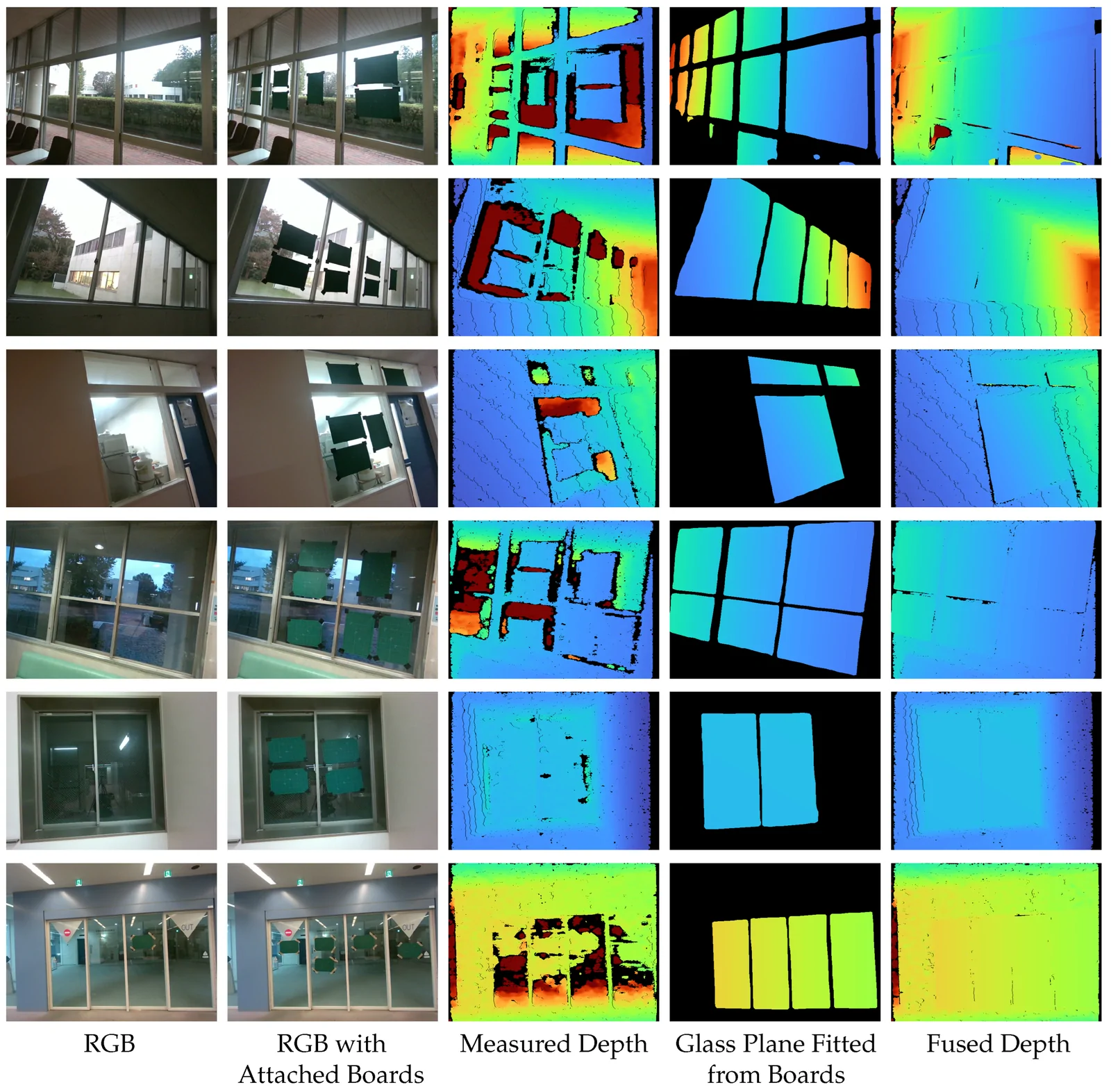
Construction of glass-depth references using opaque boards. Rows illustrate different scenes. Black denotes missing depth.

The quantitative evaluation uses 27 image pairs from eight scene groups, with each pair captured from the same fixed camera viewpoint before and after attaching opaque boards to the glass. The camera remains fixed within each pair. The opaque-board regions are manually annotated, and reference planes are jointly fitted to depth measurements within the annotated board interiors belonging to the same physical plane. These planes are then corrected for the measured 3 mm board thickness along the plane normal and projected onto the image via ray–plane intersection (Figure 9). All predictions are generated using only the bare-glass images, with a shared CGSDNet mask thresholded at 120 for the geometric methods. Board measurements and manually annotated regions are used exclusively for evaluation.

Table 3 reports Mean Absolute Error(MAE) and Root Mean Square Error (RMSE) pooled over reference-valid pixels within the shared mask where all four methods provide valid depth estimates. Coverage is computed independently over the complete predicted mask, including pixels without reference depth; it measures depth-output availability conditional on that mask, rather than segmentation recall or coverage of all physical glass surfaces. Both variants of our method have lower MAE and RMSE than the baselines on common support. The two-stage variant gives the lowest errors in this comparison. The single-stage variant groups the independently fitted block planes and uses the block plane with the most inliers as the representative of each group, so its estimate can still be affected by block-level noise or locally biased supporting measurements. The two-stage variant instead refits a common plane from the aggregated measurements of each group, reducing these local variations. This mechanism is consistent with the additional error reduction of the two-stage variant.

Coverage is slightly lower than for the baselines because glass regions with insufficient surrounding geometric support are rejected. This rejection is intentional. When the available structural depth measurements are insufficient or heavily contaminated by noise, the resulting plane estimates can become geometrically unreliable. Such estimates are discarded before being introduced into SLAM optimization, resulting in reduced coverage.

### 6.5. Qualitative Comparison of SLAM Mapping

**Figure 10:**
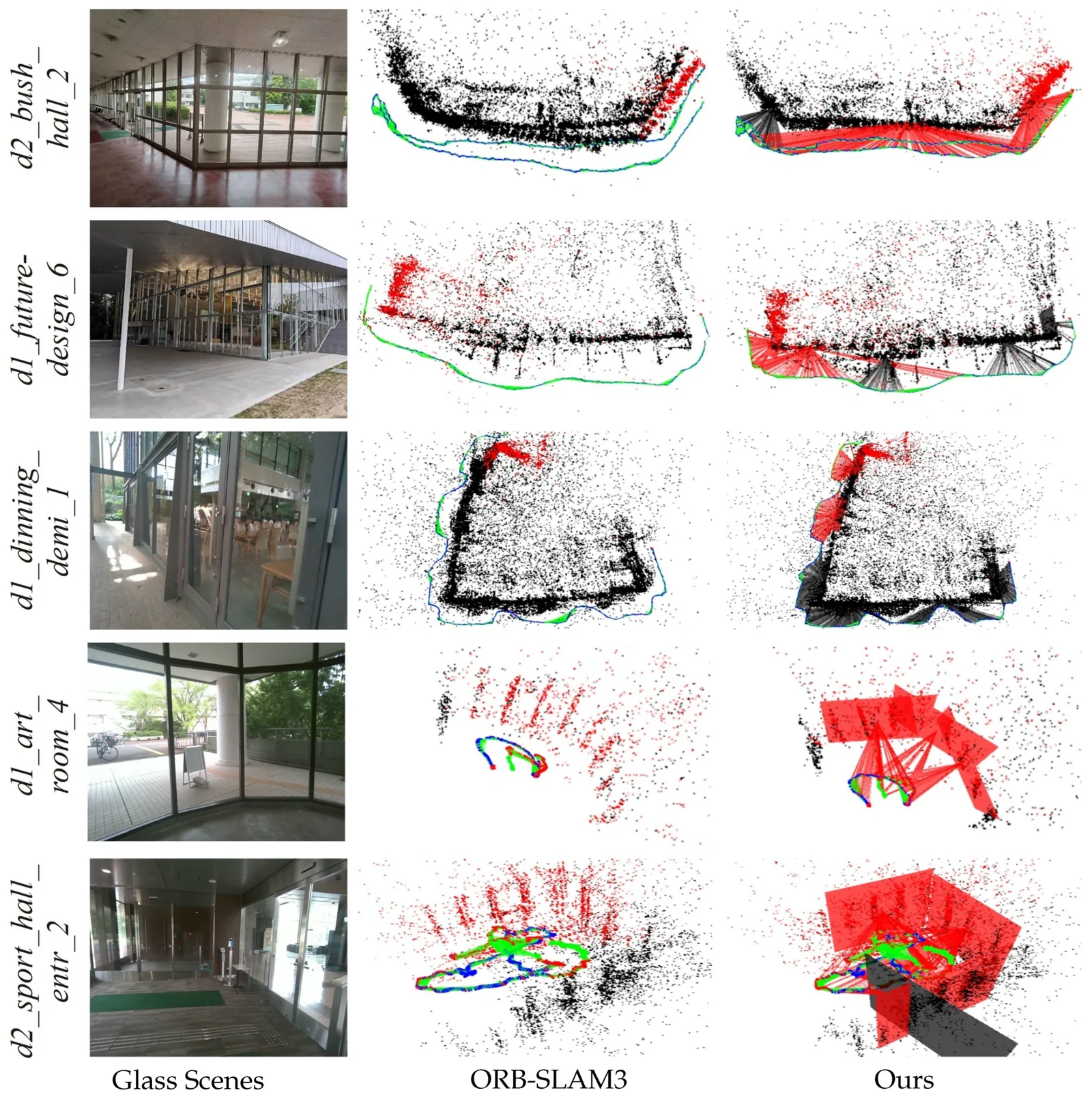
Visual comparison of SLAM mapping results between the proposed method and the baseline ORB-SLAM3 method in glass environments [4]. In our method, the estimated glass planes are highlighted by red rectangles. The keyframes observing the corresponding plane are connected to the plane center by lines.

Figure 10 presents a visual comparison of the SLAM mapping results obtained using ORB-SLAM3 [4] and our method in five representative glass scenes. The first three are large-scale scenes with long glass walls and trajectories spanning more than 10 m, while the last two are room-scale scenes.

The structures reconstructed by ORB-SLAM3 along several large glass walls appear curved or distorted. Features detected in glass regions can originate from reflections or from objects visible behind the glass, while the associated RGB-D measurements may be missing or correspond to a different physical surface. When such observations are used as ordinary point constraints, they can bias local pose estimation and gradually distort the reconstructed geometry, especially when glass occupies a large portion of the field of view over a long trajectory.

To address these failure modes, our method supplements the point constraints with glass-plane observations estimated from the surrounding structural depths. Repeated observations of the same physical glass surface are associated with a common plane landmark in local bundle adjustment, providing a persistent structural constraint even when point-level observations on the glass are unreliable. This additional plane-level geometry helps maintain the planar shape of long glass walls in Figure 10. The difference is less pronounced in room-scale scenes, where shorter trajectories limit error accumulation and surrounding non-glass objects provide enough conventional visual features for stable pose estimation.

### 6.6. Reference Trajectory Validation

**Figure 11:**
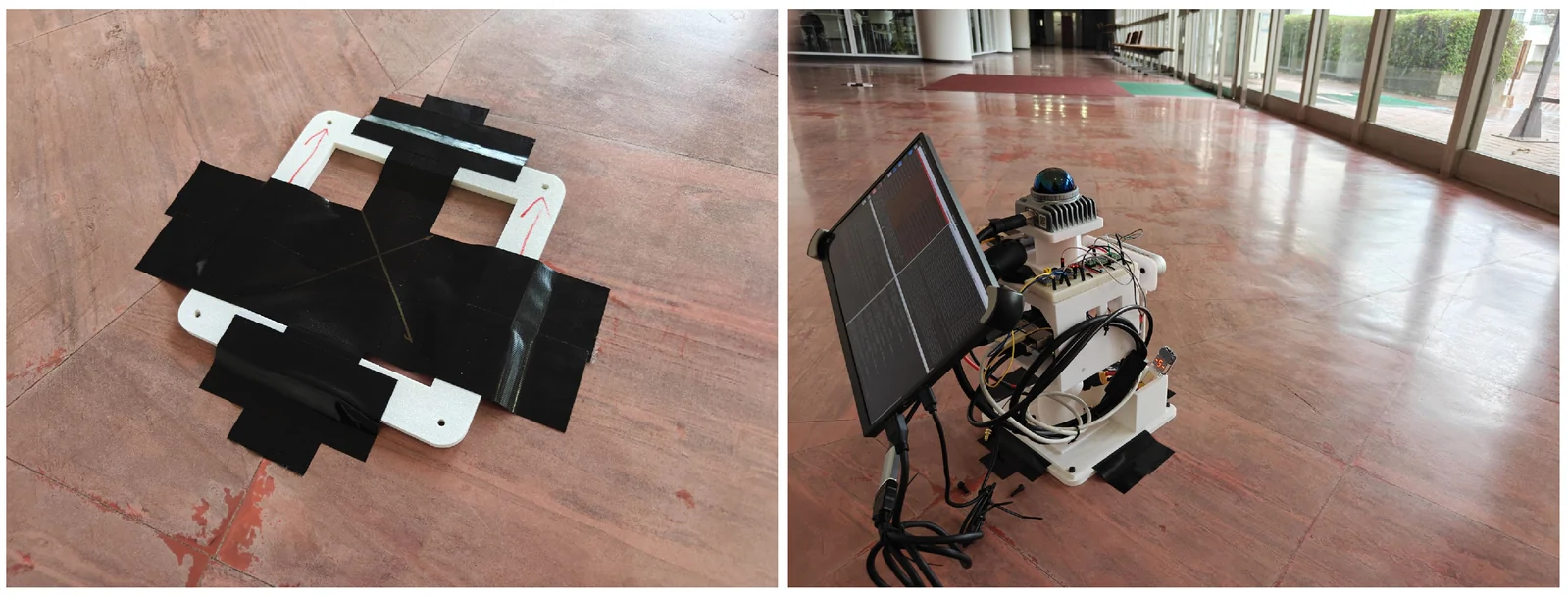
Fixed mounting plates and platform attachment. **Left**: a plate secured to the floor. **Right**: the acquisition platform fastened to the plate with four screws. The platform moves between plates; the plates remain stationary.

**Figure 12:**
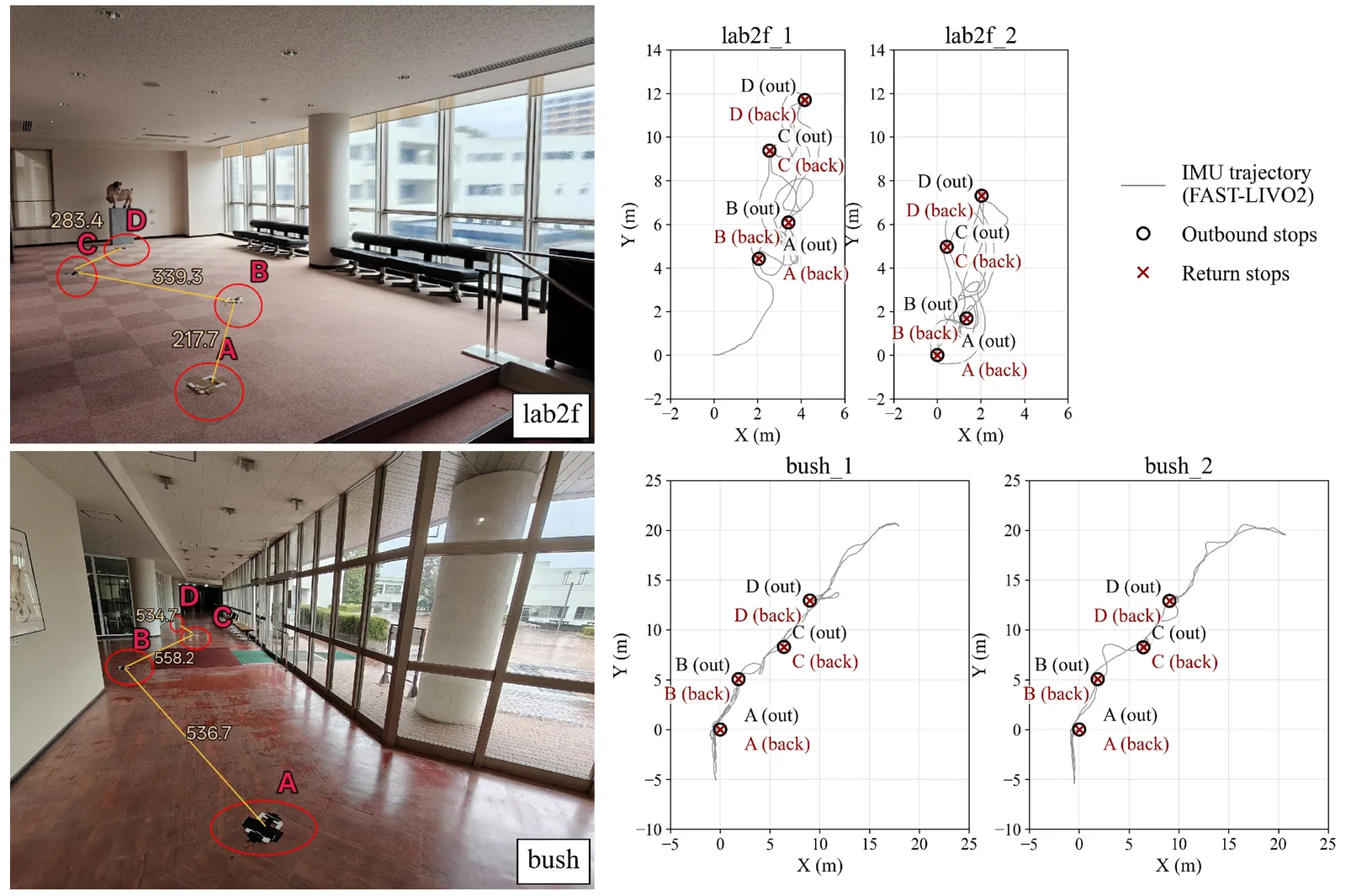
Validation scenes, stationary mounting-plate locations, tape-measured separations (unit: cm), and FAST-LIVO2 trajectories with outbound and return stops. Gray curves represent IMU trajectories; errors are evaluated at a common reference point on the platform.

We quantitatively assess the FAST-LIVO2 pseudo-reference using two recordings in each of two glass-rich scenes, lab2f and bush. Four mounting bases, labeled A–D, remain fixed to the floor throughout each recording. The acquisition platform is successively mounted on these bases in the order A–B–C–D–D–C–B–A. At each stop, the platform is secured to the corresponding base with four screws and held stationary for measurement (Figure 11). The distances between adjacent bases are independently measured with a tape, and the measured values are annotated in yellow in Figure 12. The evaluation uses two measures: (1) the horizontal discrepancy between the mean base-center positions from the two visits to the same base, and (2) the mean absolute difference between the estimated horizontal distances between adjacent bases and the corresponding tape measurements. Smaller values indicate better positional repeatability and closer agreement with the measured distances.

At each visit to a fixed mounting plate, the platform is fastened to the plate and held stationary for 3–8 s while the FAST-LIVO2 IMU poses are recorded. For evaluation, these poses are transformed to the center of the mounting base using the fixed IMU-to-base transformation. The resulting positions are averaged over each stationary interval to obtain one representative base-center position per stop. Horizontal revisit discrepancy is computed as the Euclidean distance between the mean positions obtained on the outbound and return visits to the same plate. Distance error is assessed by comparing the horizontal separation between the mean base-center positions at adjacent plates with the corresponding tape-measured distance.

Table 4 shows subcentimeter revisit discrepancies and distance errors on the order of 1–2 cm. These errors are substantially smaller than the 10–50 cm localization improvements of interest. Together with the geometric consistency discussed in Section 5.3, these measurements provide strong support for using FAST-LIVO2 trajectories as a pseudo-reference to distinguish improvements at this scale in the evaluated environments. The validation establishes local horizontal consistency; per-frame three-dimensional position and orientation errors over the full trajectories are not directly measured.

### 6.7. Quantitative Evaluation of SLAM Accuracy

We evaluate localization on all 38 sequences (18 room-scale and 20 large-scale), with each method executed 10 times per sequence. The translational Absolute Trajectory Error (ATE) RMSE is computed after rigid SE(3) alignment to the pseudo-ground-truth trajectory. Within each run, trajectory coverage is the fraction of input RGB frame timestamps with an exported camera pose, matched within 5 ms. A run satisfies the coverage requirement when this fraction is at least 90%. Separately, the run-level success rate is the number of coverage-qualified runs divided by the ten runs. Table 5 reports the median ATE RMSE together with Q1 and Q3 over coverage-qualified runs when at least six of ten runs are coverage-qualified and have evaluable ATE; otherwise, “–” and the corresponding success rate are reported. The complete systems are ORB-SLAM3 [4], ORB-SLAM2-GSD [5], PlanarSLAM [10], and our method. Ablation variants are included in the same table and analyzed separately in Section 6.8.

Differences from ORB-SLAM3 are small in most room-scale sequences and generally larger in the large-scale group. In room-scale sequences, accumulated pose error is limited and conventional features from surrounding objects often provide sufficient constraints, so the additional glass planes have less influence. In large-scale glass-rich scenes, unreliable glass-associated point observations persist over more frames and local pose errors have more opportunity to accumulate. The plane landmarks provide additional geometric constraints across local keyframes, which is consistent with the larger ATE reductions observed on several of these sequences. The quartiles show a similar pattern across repeated runs: on many large-scale sequences, the interquartile range of our method lies below or largely below that of ORB-SLAM3.

ORB-SLAM2-GSD shows strongly scene-dependent performance across both room-scale and large-scale sequences. It achieves low trajectory error on several sequences, while noticeably larger errors are observed on others. This variation may be partly related to its glass-point filtering strategy. Removing features inside detected glass regions can suppress unreliable observations caused by reflections or incorrect depth measurements. However, the same operation also reduces the number of available visual correspondences. When sufficient non-glass features remain, the system can still maintain stable pose estimation. In scenes where glass occupies a large portion of the image or the surrounding non-glass features provide limited geometric constraints, the remaining correspondences may become insufficient to constrain camera motion, leading to larger accumulated drift. The effect of glass-point filtering is examined more directly in the controlled ablation experiments in Section 6.8, where the compared configurations share the same ORB-SLAM3 backbone.

PlanarSLAM frequently fails on large-scale glass-rich sequences, particularly in long glass corridors. This behavior is consistent with the lack of stable planar constraints in such environments: missing or incorrect glass depths limit reliable plane extraction, while the remaining non-glass surfaces may provide insufficient geometric support for pose estimation.

Our method recovers glass planes from surrounding structural depth measurements and incorporates them into pose optimization while retaining point correspondences. This allows glass surfaces to provide additional constraints when their direct depth measurements are unreliable, helping reduce accumulated drift in long glass corridors. The observed benefits are more pronounced in large-scale scenes, while room-scale improvements remain less consistent.

To summarize the sequence-level results, we analyze room-scale and large-scale sequences separately.

To make each comparison fair, we include only sequences for which both our method and the baseline have reportable results. As in Table 5, each method is evaluated ten times on each sequence, and the median ATE RMSE of its valid runs is used to represent its error on that sequence.

**Figure 13:**
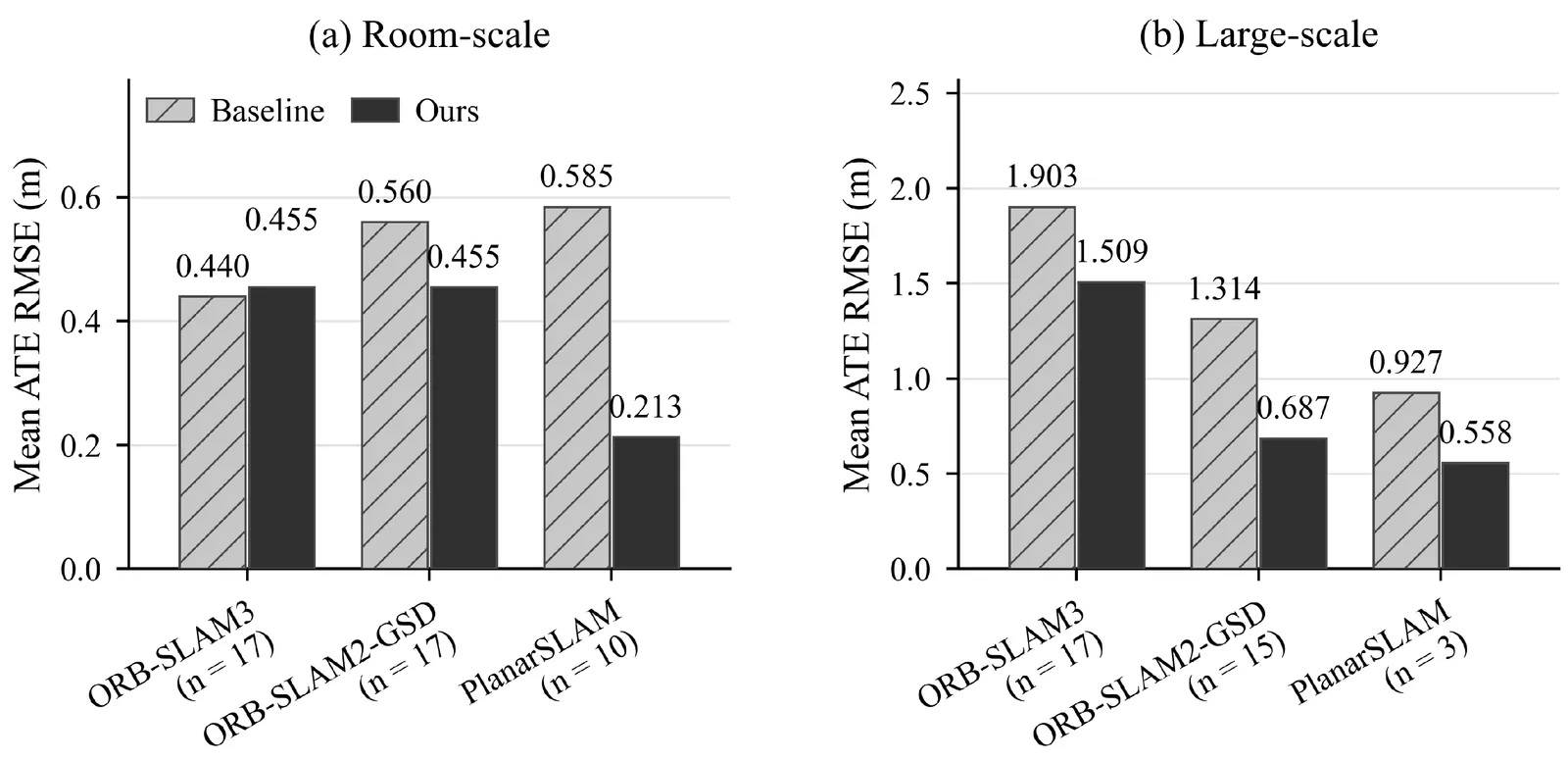
Average trajectory errors on sequences for which both methods have reportable results. Lower bars mean lower errors. Each sequence is represented by its median ATE RMSE over valid runs, and *n* is the number of sequences in the comparison. Panels (**a**) and (**b**) show room-scale and large-scale sequences, respectively.

Figure 13 then compares the average errors of the two methods across these shared sequences. Each sequence contributes one value to the average. The number of sequences is shown as *n* and can differ between baselines because some methods have reportable results on fewer sequences.

For example, on the 17 large-scale sequences shared with ORB-SLAM3, the average error is 1.903 m for ORB-SLAM3 and 1.509 m for our method. Our error is therefore lower by 0.394 m (20.7%). On the 17 room-scale sequences, the corresponding averages are 0.440 m and 0.455 m. Thus, our method does not improve the average error in this room-scale comparison.

**Figure 14:**
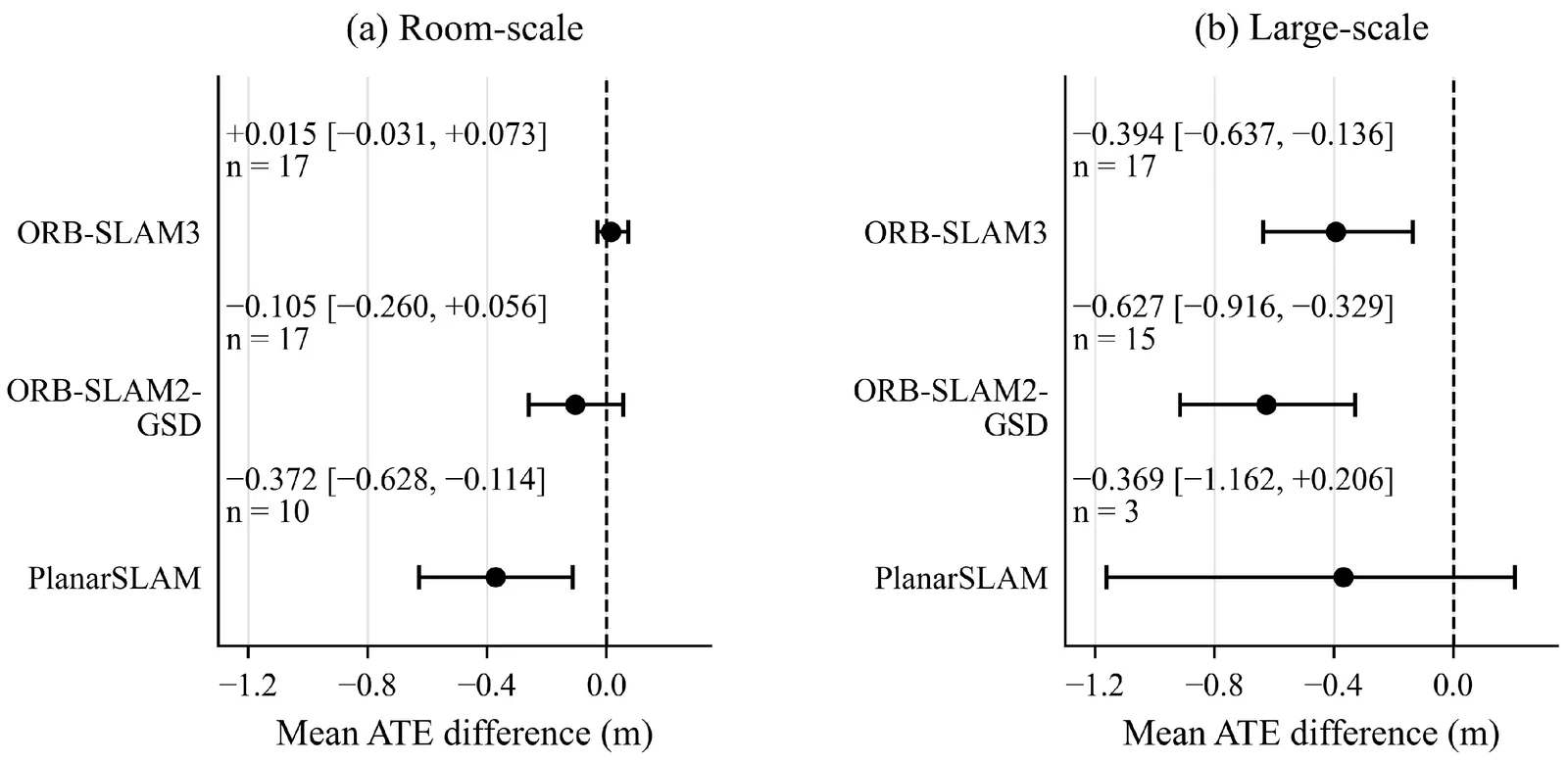
Average error difference (ours minus baseline) and its 95% bootstrap confidence interval. Each dot shows the average difference, and the horizontal line shows its uncertainty. Values to the left of zero favor our method. A line crossing zero does not clearly favor either method. An interval is calculated separately for each comparison. Panels (**a**) and (**b**) show room-scale and large-scale sequences, respectively.

Figure 14 shows the average error differences and their 95% bootstrap confidence intervals. For each shared sequence, the difference is calculated as our error minus the baseline’s error. Negative values favor our method, while positive values favor the baseline.

Each dot represents the average difference, and its horizontal line shows the confidence interval. The intervals are calculated by resampling the paired sequences 100,000 times, allowing repeats and recalculating the average difference each time. They reflect uncertainty across sequences rather than variation across repeated runs of a single sequence. An interval crossing zero does not clearly establish which method has the lower average error.

For large-scale sequences, the results favor our method over ORB-SLAM3 and ORB-SLAM2-GSD: the average errors are lower by 0.394 m and 0.627 m, respectively. For room-scale sequences, the intervals cross zero for these two baselines, so the differences do not clearly favor either method. The room-scale comparison with PlanarSLAM favors our method. For large-scale PlanarSLAM, only three sequences can be compared and the interval is wide, making this comparison less certain.

**Figure 15:**
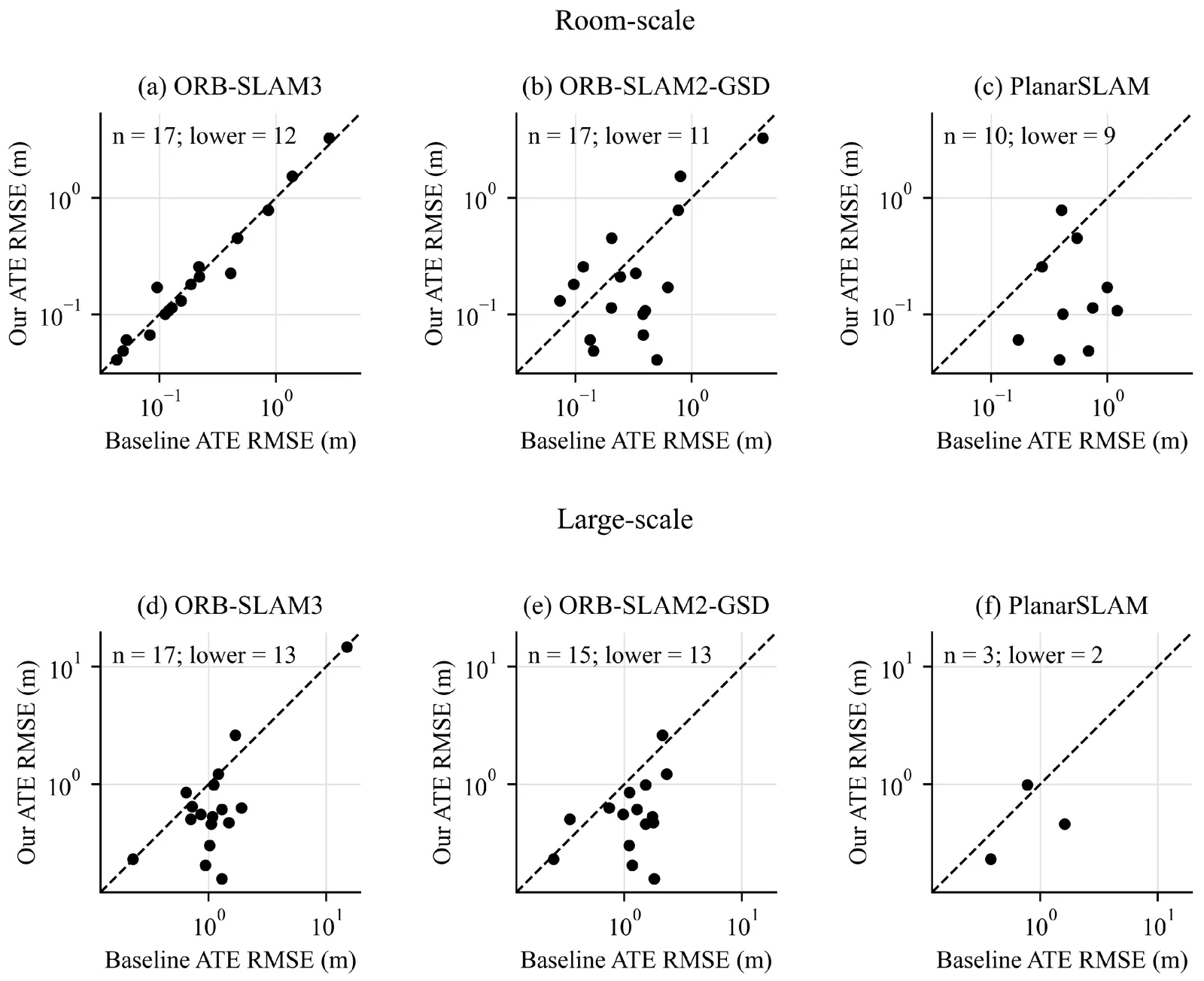
Errors on individual sequences. Each dot represents one sequence, with the baseline error on the horizontal axis and our error on the vertical axis. Both axes use logarithmic scales. The dashed line marks equal error: dots below it favor our method, and dots above it favor the baseline. Panels (**a**–**c**) and (**d**–**f**) show room-scale and large-scale sequences, respectively.

Figure 15 compares the errors on individual sequences. Each dot represents one sequence: its horizontal position is the baseline’s error, and its vertical position is our error. The diagonal line marks equal errors. A dot below the line means our method has a lower error; a dot above it means the baseline has a lower error. Dots on both sides show why a lower average error does not mean that our method is better on every sequence.

Overall, the statistical analysis indicates that the proposed method achieves lower localization errors than ORB-SLAM3 and ORB-SLAM2-GSD in large-scale scenes. In room-scale scenes, the differences from both baselines are not statistically significant.

Our method has a lower run-level success rate than ORB-SLAM3 (89.5% versus 91.1%). One possible explanation is that glass-plane landmarks are far fewer than point landmarks, with individual plane landmarks often shared by many camera poses. Consequently, a gross outlier in a plane observation may bias the shared plane estimate and affect multiple connected poses during bundle adjustment, potentially destabilizing tracking.

Large accumulated errors are still observed in some very large-scale sequences. The proposed glass-plane constraints mainly operate within local optimization and do not introduce additional global constraints over long trajectories. Their benefit depends on repeated glass-plane observations across local keyframes. Large-scale trajectories may include extended regions with little or no visible glass, limiting the availability of these additional constraints and potentially reducing the localization gains over the full trajectory. This may partly explain the varying gains across large-scale sequences. In addition, a representative loop-closing sequence is discussed separately in Section 6.9 to illustrate the complementary roles of local glass-plane constraints and global loop correction.

### 6.8. Ablation Experiments

We conduct ablation experiments to investigate three aspects of the proposed system: (1) the effect of glass-point filtering, (2) the contribution of incorporating glass-plane constraints into bundle adjustment (BA), and (3) the effect of the proposed two-stage RANSAC for glass-plane estimation. The corresponding configurations are summarized in Table 6. The effect of glass-point filtering is examined through two controlled comparisons, ORB-SLAM3 versus Ablation1 and Ablation2 versus our complete method. The contribution of glass-plane BA is evaluated by comparing ORB-SLAM3 with our method and Ablation1 with Ablation2. Finally, Ablation3 and our complete method share the same glass-plane BA formulation and block grouping, but use a representative block plane and a plane refitted from aggregated measurements, respectively, allowing us to evaluate the effect of the second-stage refitting. All ablations use the coverage and ATE reporting criteria defined in Section 6.7.

First, glass-point filtering shows no consistent localization benefit and can increase trajectory error. In both controlled comparisons, retaining glass-region point correspondences often leads to lower ATE, although the effect varies across scenes. This suggests that mask-based filtering discards useful visual information along with unreliable observations, reducing the constraints available for pose estimation.

Second, incorporating glass-plane BA reduces ATE on many sequences, both with and without glass-point filtering. The recovered glass surfaces provide useful structural constraints in addition to point features, particularly where repeated glass observations can limit local drift. This supports the contribution of glass-plane geometry to pose optimization within the common SLAM backbone.

Output availability provides a complementary assessment. Several difficult sequences yield reportable ATE for Ablation1 but not Ablation2; similarly, some large-scale sequences satisfy the reporting criterion for ORB-SLAM3 but not the complete method. These outcomes are consistent with the risk that inaccurate or weakly constrained plane estimates adversely affect optimization, emphasizing the need for reliable plane estimation.

Finally, comparison with Ablation3 shows that the two-stage procedure often reduces ATE in both room-scale and large-scale scenes. In several improved cases, the quartiles also shift toward lower errors across repeated runs. This pattern is consistent with aggregation and refitting reducing the influence of locally biased block planes and producing more consistent geometric constraints. Some sequences still show degradation, so the second stage does not provide a uniform improvement.

The ablation experiments support three conclusions. Glass-point filtering generally increases trajectory error, suggesting that useful visual correspondences are also discarded. Glass-plane BA shows a clear trend toward lower trajectory error, supporting the use of recovered glass geometry for pose estimation. Two-stage RANSAC further reduces ATE relative to single-stage estimation in multiple sequences, including substantial improvements in both room-scale and large-scale scenes. The complete system therefore retains glass-region point observations and supplements them with robustly estimated glass-plane constraints.

Additionally, though identifying the optimal value of λ is beyond the scope of this study, to examine how the choice of λ affects performance, we conducted a sensitivity analysis of the plane-constraint weight in Section 4.6 using two room-scale and two large-scale sequences (Table 7). The tested values are λ∈{1,100,500,1000,5000}, with all other parameters kept fixed. The λ=1000 results are taken from the original ten-run experiments, while the other settings use additional batches with five planned runs each. Medians and quartiles summarize valid runs.

The sensitivity results show that the effect of λ is strongly scene-dependent, with different scenes favoring different weights. As λ controls the balance between point and plane constraints, this variation may reflect differences in the available point constraints and the reliability of the recovered glass planes. A larger weight can strengthen useful geometric constraints, but can also increase the influence of plane-estimation errors. The main and ablation experiments demonstrate substantial localization gains in multiple glass scenes using a fixed weight across all sequences. Together, these results support the value of incorporating glass-plane geometry and motivate weighting its contribution according to observation quality in future work.

### 6.9. Qualitative Analysis of Loop Closing

In a loop-closing sequence, the camera returns to a previously observed region, allowing accumulated trajectory drift to be corrected once a loop is detected. Our method retains the loop-closing and global optimization mechanisms of ORB-SLAM3 [4]. We examine how the proposed local glass-plane constraints operate together with this existing mechanism by comparing the reconstructed maps before and after loop correction.

**Figure 16:**
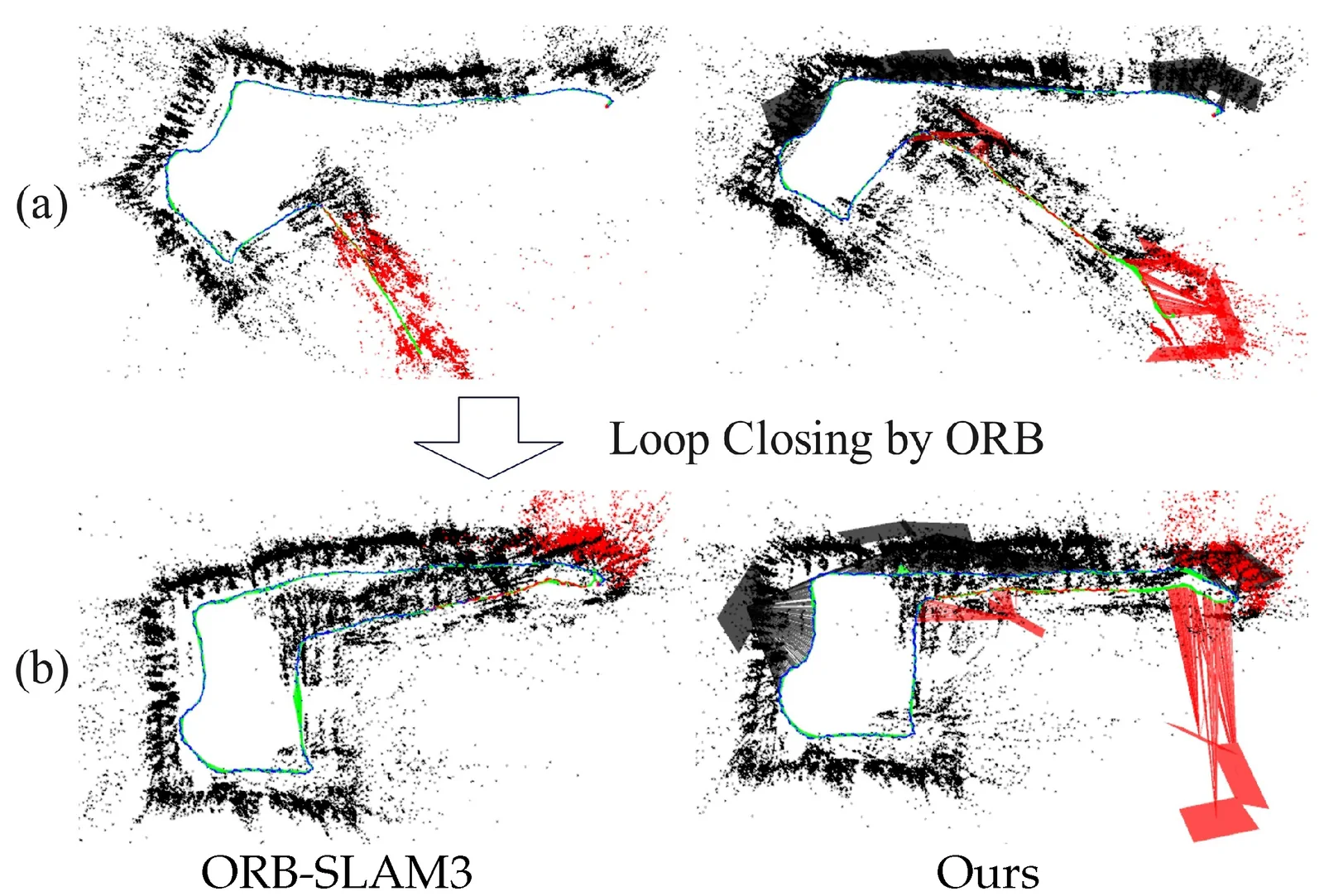
Comparison between ORB-SLAM3 and our method on a loop-closing sequence. (**a**) Mapping results before loop closure detection. (**b**) Mapping results after loop closure detection and correction. Our method uses the original loop-detection module of ORB-SLAM3 [4].

Figure 16 shows the results on d2_dining_kasuga_inside_wide. The camera starts near the entrance of the cafeteria, moves through the interior area over a long trajectory, and finally returns to the starting region. Before loop closure, both ORB-SLAM3 and our method exhibit accumulated global drift. The additional glass-plane constraints provide local geometric information around large glass surfaces and help maintain the consistency of the reconstructed structure before loop closure.

When the camera returns to the previously observed region and a loop is successfully detected, the loop-closing module inherited from ORB-SLAM3 performs global correction of the accumulated trajectory error. As shown in Figure 16, the reconstructed point map becomes globally more consistent after loop correction. This example illustrates the correction of accumulated drift by the inherited loop-closing mechanism, while the glass-plane constraints contribute through local optimization.

This loop-closing example illustrates the complementary roles of the two mechanisms. Glass-plane constraints support local pose estimation and geometric consistency before loop closure, while loop closing corrects accumulated drift after a previously visited region is recognized. The proposed method therefore combines local geometric constraints from glass surfaces with the global correction provided by the existing loop-closing mechanism.

### 6.10. Qualitative Comparison of Dense Point Cloud Reconstruction

**Figure 17:**
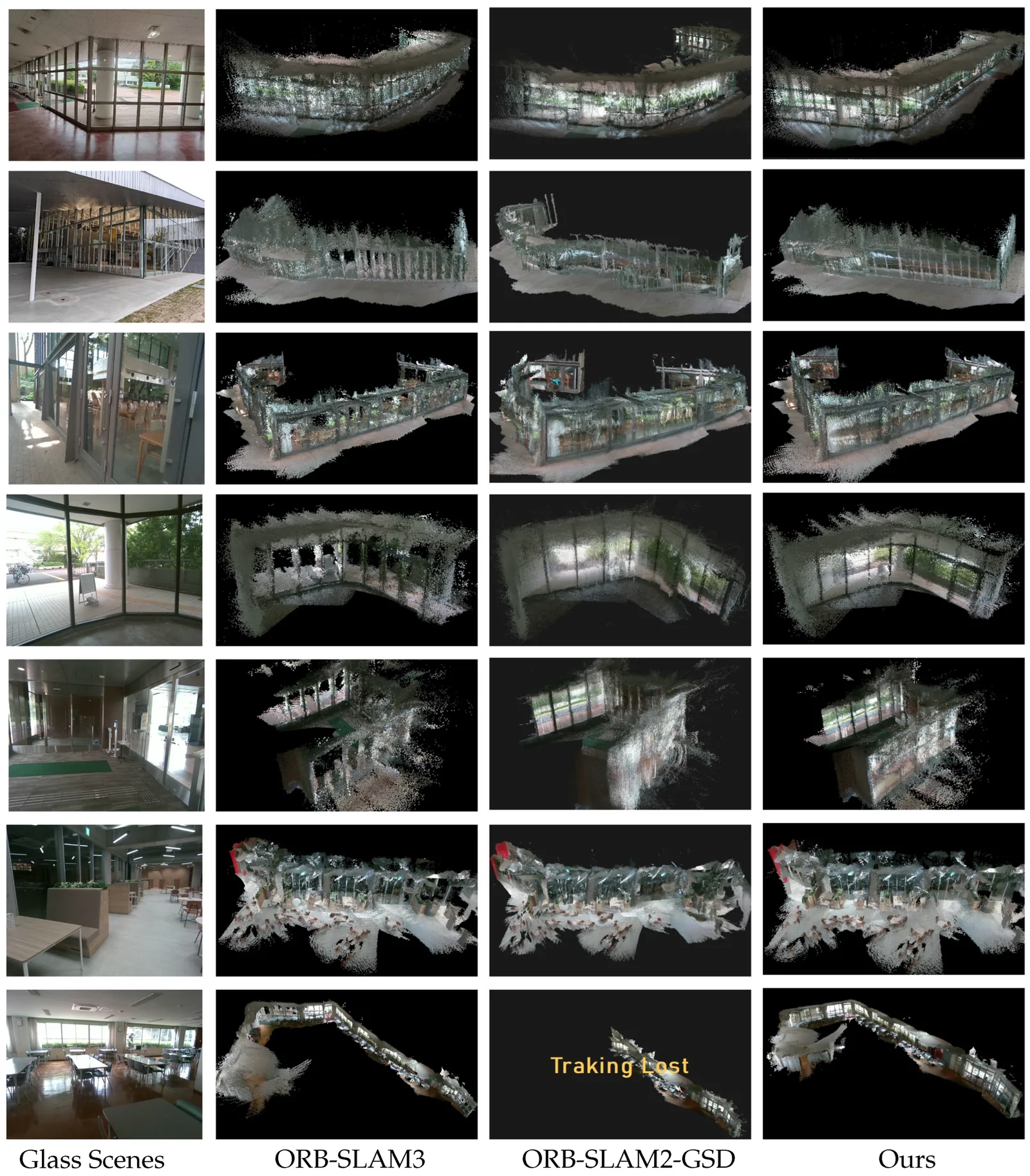
Visual comparison of dense reconstruction results on the UT-SRL Glass RGB-D Dataset [4,5].

We also evaluate dense point cloud reconstruction in glass scenes. While the previous experiments focus on camera trajectory accuracy and SLAM map consistency, this experiment further examines how these improvements affect dense reconstruction. We compare ORB-SLAM3 [4], ORB-SLAM2-GSD [5], and our proposed method. Figure 17 shows the dense point cloud reconstruction results of the three methods in representative glass scenes.

As shown in Figure 17, ORB-SLAM3 fails to reconstruct glass surfaces in dense mapping. Since raw RGB-D measurements on glass are often missing or invalid, glass regions cannot be converted into reliable 3D points. As a result, large holes appear in the dense point clouds at the locations of glass surfaces. This indicates that a standard RGB-D SLAM pipeline cannot directly recover glass geometry from the original depth images.

ORB-SLAM2-GSD can reconstruct glass surfaces to some extent by estimating depths for glass regions. However, the reconstructed glass surfaces remain geometrically inconsistent in several examples. The estimated regions often form uneven point sets, and some areas contain clearly incorrect depths. Local depth inconsistencies produced by Four-Directional Depth can contribute to this uneven geometry. Pose-estimation errors in large-scale scenes may further introduce misalignment and distortion into the accumulated point cloud.

Our method produces visually more consistent dense reconstructions in these examples. The estimated glass regions form smoother and more planar surfaces, and the accumulated point clouds exhibit better geometric alignment. These qualitative results illustrate how the recovered glass geometry contributes to dense reconstruction.

### 6.11. Runtime Experiments

We evaluate the runtime of the glass depth estimation module on the d2_bush_hall_1 sequence. As shown in Table 8, CGSDNet-Depth requires 36.47 ms/frame on a GPU, while Four-Directional Depth and our method require 43.92 ms/frame and 77.43 ms/frame on a CPU, respectively. Because the methods are executed on different devices, these measurements characterize the practical computational cost of the current implementations and should not be interpreted as a hardware-normalized runtime comparison. The runtime breakdown of our method is reported in Table 9. The RANSAC filtering and merging stage is the most time-consuming component, requiring 58.21 ms and accounting for approximately 75% of the total runtime.

In the current implementation, glass-surface estimation is performed offline, and real-time operation is outside the scope of this study. The reported runtime therefore characterizes the computational cost of the depth-estimation module. Since glass-plane estimation is required only for selected keyframes, an online implementation could reduce the average computational load by invoking the module at keyframe level. Further optimization of the RANSAC-based processing remains necessary for real-time deployment.

## 7. Conclusions

In this paper, we investigated the explicit use of glass-surface geometry as additional constraints for RGB-D SLAM localization in glass-rich environments. The proposed GlassPlane-SLAM uses a two-stage RANSAC-based procedure to recover unified glass planes from noisy metric depth measurements of approximately coplanar surrounding structures, reducing the influence of unreliable local observations. The recovered planes are associated with shared landmarks across keyframes and jointly optimized with conventional point landmarks and camera poses in local bundle adjustment. This allows glass surfaces themselves to provide additional geometric constraints for camera pose estimation while useful point correspondences are preserved.

We also constructed the UT-SRL Glass RGB-D Dataset, containing 38 room-scale and large-scale sequences for evaluating SLAM in glass-rich environments. Experimental results show lower trajectory errors on many sequences, with particularly pronounced gains in several large-scale scenes. Paired statistical comparisons further support lower mean ATE than ORB-SLAM3 and ORB-SLAM2-GSD on the evaluated large-scale subsets, while the room-scale confidence intervals do not establish a clear mean improvement over these two baselines. Qualitative results further show improved geometric consistency in the resulting maps, supporting the effectiveness of the proposed glass-plane constraints for localization in glass-rich environments.

## Figures and Tables

**Table 1:** Characteristics of the 38 evaluated sequences from 17 acquisition scene groups. Scene IDs retain the author-defined acquisition groups and are numbered by first appearance.

Dataset	Scene ID	Duration (s)	Reference Path (m)	Glass-Pixel Ratio	Glass Planes	Loop Detected ^†^	Lighting
Room-scale sequences							
d1_art_room_1	G01	32.7	7.72	64.2%	5	–	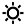
d1_art_room_2	G01	39.9	6.70	58.9%	5	–	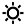
d1_art_room_3	G01	15.1	3.59	63.4%	5	–	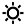
d1_art_room_4	G01	30.8	9.14	73.2%	5	–	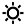
d1_doom_hall_1f_1	G02	50.7	15.74	27.0%	3	–	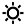
d1_doom_hall_1f_2	G02	44.1	19.71	20.8%	4	–	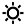
d1_sports_room_in_1	G03	73.6	18.77	52.4%	4	–	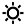
d1_sports_room_in_2	G03	38.4	10.34	40.2%	3	–	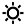
d1_sports_room_in_3	G03	22.9	5.15	44.0%	3	–	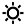
d1_sports_room_out_1	G04	57.8	22.16	56.7%	6	–	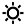
d1_sports_room_out_2	G04	69.2	25.13	53.2%	6	–	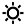
d2_dining_entr	G05	166.3	39.72	35.2%	5	–	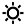
d2_sport_hall_entr_1	G06	121.7	24.78	53.1%	3	–	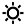
d2_sport_hall_entr_2	G06	154.8	34.13	51.0%	3	–	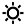
d2_student_space	G07	150.6	32.33	36.2%	6	–	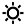
d3_futuredesign_room1_loop1	G08	230.3	58.01	35.0%	2	✔	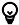
d3_futuredesign_room1_loop2	G08	169.3	47.85	25.9%	2	✔	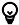
d3_futuredesign_room2	G09	181.4	39.99	44.6%	3	–	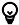
Large-scale sequences							
d1_dining_demi_1	G10	215.2	71.48	48.5%	4	–	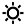
d1_dining_demi_2	G10	276.8	103.43	52.0%	4	–	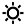
d1_doom_hall_2f_1	G11	258.7	67.92	48.8%	2	–	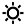
d1_doom_hall_2f_2	G11	96.8	50.83	37.8%	2	–	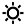
d1_futuredesign_1	G12	110.1	35.35	54.5%	4	–	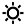
d1_futuredesign_2	G12	174.2	65.49	58.6%	4	–	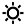
d1_futuredesign_3	G12	136.7	64.26	53.4%	6	–	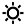
d1_futuredesign_4	G10	121.4	51.46	51.7%	3	–	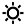
d1_futuredesign_5	G12	182.0	56.34	52.3%	6	–	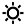
d1_futuredesign_6	G12	64.4	19.81	62.9%	4	–	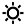
d2_bush_hall_1	G13	190.5	52.70	54.4%	3	–	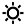
d2_bush_hall_2	G13	342.0	94.51	56.9%	3	–	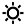
d2_dining_kasuga_inside	G14	205.7	68.51	32.4%	3	–	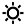
d2_dining_kasuga_inside_wide	G14	274.3	91.65	23.2%	4	✔	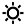
d3_futuredesign_1f_coffee	G15	210.6	71.01	41.2%	2	–	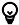
d3_futuredesign_1fhall_1	G16	209.8	52.53	65.4%	6	–	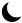
d3_futuredesign_1fhall_2	G16	214.7	47.80	60.3%	6	–	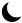
d3_futuredesign_1fhall_loop	G16	317.7	83.00	76.6%	6	–	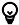
d3_futuredesign_2fto1f	G17	163.1	52.20	66.9%	2	–	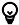
d3_futuredesign_2fto1f_wide	G17	296.5	130.98	54.8%	4	–	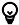

Notes: Duration is the elapsed time between the first and last input timestamps. Reference path is the cumulative length of the available FAST-LIVO2 pseudo-reference trajectory. The predicted glass pixel ratio is the mean per-frame fraction of pixels with saved 8-bit mask values ≥ 120. Physical glass-plane counts are manually annotated. ^†^ ✔ indicates that the ORB-SLAM3 loop-closing module detected a loop in at least 8 out of 10 runs, while – indicates fewer than 8 runs. Lighting: 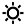 diffuse daylight without direct sunlight; 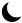 dim natural light at dusk; 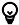 artificial lighting at night. Lighting categories are qualitative author annotations.

**Table 2:** Glass-mask accuracy on 51 manually annotated images from 17 acquisition scenes. Metrics are computed by pooling pixels across all images at the threshold used for contour and window-frame extraction.

Mask Threshold	Glass IoU	Precision	Recall
>120	87.19%	89.73%	96.85%

**Table 3:** Glass-depth estimation relative to reference planes obtained using opaque boards on 27 image pairs. MAE and RMSE use common valid pixels; coverage uses the complete shared predicted mask. The lowest MAE and RMSE values are shown in bold.

Method	MAE (m)	RMSE (m)	Coverage (%)
Four-Directional Depth	0.2279	1.1230	99.98
CGSDNet-Depth	0.4886	0.6208	100.00
Ours (Single-Stage RANSAC)	0.0590	0.1022	95.46
Ours (Two-Stage RANSAC)	**0.0456**	**0.0686**	95.01

**Table 4:** Validation of the pseudo-reference trajectories. Both revisit discrepancy and distance error are expressed in centimeters. The overall results are computed from 16 paired stops and 24 distance comparisons from four recordings.

	Revisit Discrepancy (cm)		Distance Error (cm)	
Sequence	Mean	Max	MAE	RMSE
lab2f_1	0.211	0.320	1.38	1.56
lab2f_2	0.192	0.251	1.28	1.34
bush_1	0.218	0.670	1.06	1.22
bush_2	0.117	0.150	1.29	1.44
Overall	0.184	0.670	1.25	1.39

**Table 5:** ATE RMSE of camera trajectories over 10 runs for each sequence, reported as median [Q1, Q3] over coverage-qualified runs. ATE statistics are reported only when at least six of the ten runs meet the coverage requirement and have evaluable ATE; otherwise, “– (n/10)” is shown, where *n* is the number of coverage-qualified runs; this count is distinct from the within-run trajectory coverage. The **numerically lowest** and second-lowest valid ATE results are highlighted.

Dataset	ORB-SLAM3 [4]	ORB-SLAM2-GSD [5]	PlanarSLAM [10]	Ablation1	Ablation2	Ablation3	Ours
Room-scale sequences							
d1_art_room_1	0.112 [0.103, 0.121]	0.382 [0.369, 0.391]	0.414 [0.400, 0.430]	0.271 [0.270, 0.287]	0.218 [0.207, 0.223]	0.110 [0.109, 0.113]	**0.100 [0.098, 0.103]**
d1_art_room_2	0.043 [0.042, 0.045]	0.504 [0.494, 0.508]	0.388 [0.376, 0.421]	0.348 [0.337, 0.363]	0.268 [0.257, 0.284]	0.050 [0.048, 0.050]	**0.040 [0.039, 0.042]**
d1_art_room_3	0.049 [0.043, 0.050]	0.144 [0.132, 0.154]	0.688 [0.685, 0.711]	0.118 [0.109, 0.138]	0.114 [0.111, 0.117]	0.050 [0.047, 0.054]	**0.048 [0.047, 0.049]**
d1_art_room_4	0.120 [0.107, 0.132]	0.399 [0.363, 0.419]	1.215 [1.189, 1.239]	0.219 [0.205, 0.231]	0.134 [0.116, 0.176]	0.118 [0.111, 0.125]	**0.107 [0.104, 0.115]**
d1_doom_hall_1f_1	0.128 [0.119, 0.133]	0.204 [0.164, 0.212]	0.747 [0.622, 0.793]	0.187 [0.183, 0.194]	0.181 [0.151, 0.202]	0.154 [0.147, 0.192]	**0.113 [0.110, 0.142]**
d1_doom_hall_1f_2	0.221 [0.177, 0.253]	0.244 [0.233, 0.267]	– (1/10)	0.219 [0.196, 0.321]	0.119 [0.115, 0.122]	**0.110 [0.100, 0.132]**	0.210 [0.198, 0.217]
d1_sports_room_in_1	**0.096 [0.080, 0.134]**	0.624 [0.606, 0.654]	0.996 [0.973, 1.008]	0.481 [0.473, 0.490]	0.201 [0.192, 0.209]	0.170 [0.162, 0.202]	0.170 [0.142, 0.191]
d1_sports_room_in_2	0.154 [0.135, 0.197]	**0.074 [0.068, 0.087]**	– (3/10)	0.548 [0.146, 0.552]	0.551 [0.070, 0.553]	0.129 [0.124, 0.149]	0.130 [0.123, 0.142]
d1_sports_room_in_3	0.052 [0.050, 0.054]	0.134 [0.129, 0.139]	0.172 [0.166, 0.178]	0.075 [0.071, 0.079]	**0.052 [0.048, 0.057]**	0.073 [0.073, 0.078]	0.060 [0.055, 0.072]
d1_sports_room_out_1	0.187 [0.168, 0.200]	**0.097 [0.094, 0.101]**	– (3/10)	0.208 [0.187, 0.238]	0.140 [0.126, 0.141]	0.210 [0.192, 0.226]	0.180 [0.162, 0.200]
d1_sports_room_out_2	0.218 [0.212, 0.230]	**0.117 [0.112, 0.120]**	0.274 [0.272, 0.285]	0.234 [0.204, 0.260]	0.197 [0.187, 0.212]	0.255 [0.238, 0.276]	0.256 [0.211, 0.272]
d2_dining_entr	– (4/10)	**0.270 [0.260, 0.281]**	0.676 [0.674, 0.687]	– (4/10)	– (4/10)	– (3/10)	– (2/10)
d2_sport_hall_entr_1	0.083 [0.080, 0.085]	0.384 [0.351, 0.389]	– (1/10)	0.215 [0.203, 0.224]	0.133 [0.104, 0.140]	0.072 [0.063, 0.077]	**0.066 [0.066, 0.073]**
d2_sport_hall_entr_2	0.410 [0.349, 0.433]	0.331 [0.296, 0.358]	– (0/10)	0.607 [0.581, 0.621]	0.444 [0.438, 0.469]	**0.208 [0.169, 0.370]**	0.225 [0.081, 0.250]
d2_student_space	0.863 [0.835, 0.871]	0.769 [0.732, 0.794]	**0.404 [0.392, 0.413]**	0.955 [0.864, 1.031]	0.679 [0.541, 0.782]	0.795 [0.626, 0.816]	0.783 [0.593, 0.829]
d3_futuredesign_room1_loop1	**2.881 [2.465, 5.346]**	4.101 [3.794, 4.599]	– (5/10)	4.988 [4.800, 5.539]	– (3/10)	– (5/10)	3.258 [2.817, 3.639]
d3_futuredesign_room1_loop2	0.469 [0.364, 0.510]	**0.206 [0.199, 0.576]**	0.548 [0.455, 1.898]	0.367 [0.322, 0.415]	1.218 [1.136, 1.304]	1.112 [0.901, 1.207]	0.451 [0.415, 0.493]
d3_futuredesign_room2	1.388 [1.251, 1.571]	0.801 [0.787, 0.823]	– (2/10)	2.049 [1.646, 2.548]	**0.564 [0.529, 0.619]**	1.472 [1.007, 1.619]	1.535 [1.449, 1.560]
Large-scale sequences							
d1_dining_demi_1	1.213 [1.031, 1.269]	2.295 [2.241, 2.358]	– (0/10)	3.906 [3.440, 4.463]	1.297 [1.281, 1.390]	**0.568 [0.543, 0.637]**	1.217 [1.096, 1.405]
d1_dining_demi_2	– (0/10)	0.927 [0.842, 0.995]	– (0/10)	1.118 [1.063, 1.208]	**0.509 [0.485, 0.629]**	0.553 [0.497, 0.687]	– (4/10)
d1_doom_hall_2f_1	0.726 [0.703, 0.757]	– (1/10)	– (0/10)	1.815 [1.794, 1.844]	– (4/10)	0.698 [0.689, 0.712]	**0.645 [0.616, 0.685]**
d1_doom_hall_2f_2	0.707 [0.645, 0.769]	**0.344 [0.317, 0.872]**	– (4/10)	1.071 [0.962, 1.189]	– (5/10)	0.468 [0.440, 0.505]	0.503 [0.465, 0.538]
d1_futuredesign_1	0.942 [0.924, 0.985]	1.169 [1.156, 1.180]	– (2/10)	1.228 [1.217, 1.266]	0.446 [0.408, 0.487]	0.348 [0.335, 0.368]	**0.204 [0.194, 0.225]**
d1_futuredesign_2	1.491 [1.419, 1.496]	1.766 [1.744, 1.814]	– (0/10)	1.790 [1.767, 1.805]	0.488 [0.457, 0.552]	**0.443 [0.425, 0.465]**	0.470 [0.462, 0.488]
d1_futuredesign_3	**0.646 [0.631, 0.669]**	1.107 [1.077, 1.208]	– (0/10)	0.742 [0.734, 0.756]	0.905 [0.870, 1.001]	0.900 [0.849, 0.979]	0.850 [0.623, 0.953]
d1_futuredesign_4	1.110 [0.827, 1.147]	1.519 [1.293, 1.557]	**0.779 [0.686, 0.824]**	1.948 [1.116, 2.295]	0.888 [0.817, 0.976]	1.067 [0.953, 1.257]	0.986 [0.915, 1.093]
d1_futuredesign_5	1.301 [1.286, 1.308]	1.282 [1.253, 1.284]	– (1/10)	1.260 [1.254, 1.291]	0.711 [0.660, 0.723]	0.868 [0.774, 0.964]	**0.609 [0.579, 0.706]**
d1_futuredesign_6	0.228 [0.220, 0.243]	0.250 [0.242, 0.253]	0.380 [0.251, 0.387]	0.228 [0.221, 0.231]	**0.196 [0.194, 0.200]**	0.218 [0.214, 0.223]	0.230 [0.224, 0.243]
d2_bush_hall_1	1.300 [1.262, 1.329]	1.797 [1.752, 1.851]	– (0/10)	1.802 [1.782, 1.818]	0.213 [0.192, 0.240]	0.437 [0.428, 0.459]	**0.157 [0.149, 0.168]**
d2_bush_hall_2	1.023 [0.873, 1.116]	1.101 [1.066, 1.278]	– (0/10)	1.666 [1.554, 1.747]	**0.288 [0.272, 0.320]**	0.877 [0.726, 1.079]	0.301 [0.226, 0.421]
d2_dining_kasuga_inside	1.083 [1.042, 1.094]	1.735 [1.693, 1.762]	– (5/10)	1.646 [1.634, 1.659]	0.549 [0.485, 0.673]	**0.447 [0.407, 0.463]**	0.529 [0.512, 0.558]
d2_dining_kasuga_inside_wide	1.056 [1.047, 1.136]	1.519 [1.179, 1.996]	1.620 [1.051, 2.138]	1.229 [1.225, 1.241]	6.492 [4.482, 6.547]	1.222 [0.967, 1.589]	**0.458 [0.437, 0.553]**
d3_futuredesign_1f_coffee	0.860 [0.797, 0.916]	0.976 [0.938, 1.028]	– (3/10)	0.945 [0.838, 1.032]	1.450 [0.621, 1.510]	1.547 [1.362, 1.709]	**0.553 [0.418, 0.820]**
d3_futuredesign_1fhall_1	1.912 [1.629, 2.163]	0.744 [0.724, 0.839]	– (2/10)	7.306 [6.572, 8.071]	– (2/10)	1.781 [1.537, 1.813]	**0.628 [0.377, 0.937]**
d3_futuredesign_1fhall_2	**1.002 [0.922, 1.121]**	– (2/10)	– (0/10)	6.780 [5.399, 9.055]	8.750 [8.466, 9.454]	1.206 [0.927, 1.296]	– (5/10)
d3_futuredesign_1fhall_loop	**6.437 [6.032, 7.372]**	– (0/10)	– (0/10)	9.030 [8.837, 9.436]	– (3/10)	– (2/10)	– (4/10)
d3_futuredesign_2fto1f	1.692 [0.964, 4.838]	2.112 [2.020, 2.190]	– (0/10)	2.439 [1.971, 2.929]	**1.500 [1.418, 1.712]**	2.241 [2.113, 2.384]	2.612 [1.931, 2.661]
d3_futuredesign_2fto1f_wide	15.067 [13.588, 16.544]	– (1/10)	– (0/10)	15.625 [15.331, 16.637]	**2.082 [1.926, 3.098]**	4.972 [4.157, 6.628]	14.707 [7.116, 15.731]

**Table 6:** Ablation study of the proposed components.

Method	Glass-Point Filtering	Glass-Plane BA	Two-Stage RANSAC
ORB-SLAM3 (Baseline)	-	-	-
Ablation1	✔	-	-
Ablation2	✔	✔	✔
Ablation3	-	✔	-
Ours	-	✔	✔

**Table 7:** Sensitivity to the plane-constraint weight: ATE RMSE reported as median [Q1, Q3] (m).

Sequence	λ=1	100	500	1000 ^†^	5000
Room-scale					
d2_sport_hall_entr_1	0.085 [0.078,0.092]	0.088 [0.085,0.088]	0.073 [0.066,0.079]	0.066 [0.066,0.073]	0.141 [0.138,0.147]
d1_sports_room_in_1	0.082 [0.080,0.099]	0.120 [0.101,0.141]	0.163 [0.149,0.183]	0.170 [0.142,0.191]	0.236 [0.226,0.304]
Large-scale					
d2_bush_hall_1	1.172 [1.153,1.226]	0.576 [0.521,0.629]	0.242 [0.222,0.259]	0.157 [0.149,0.168]	0.270 [0.254,0.279]
d1_futuredesign_3	0.692 [0.606,0.715]	0.806 [0.781,0.821]	0.899 [0.745,0.981]	0.850 [0.623,0.953]	0.808 [0.612,1.022]

^†^ The λ=1000 column reproduces the original ten-run experiments. The other columns use additional batches with five planned runs per setting. Values are median [Q1, Q3] ATE RMSE over valid runs. The valid-run counts, listed in the order λ=1/100/500/5000, are 5/3/5/5 for the first sequence, 5/5/5/5 for the second, 4/5/4/5 for the third, and 5/5/5/5 for the fourth.

**Table 8:** Runtime comparison of glass surface depth estimation methods. The downward arrow indicates that lower values are better.

Method	Execution Device	Runtime (ms/Frame) ↓
CGSDNet-Depth	GPU	36.47
Four-Directional Depth	CPU	43.92
Ours	CPU	77.43

**Table 9:** Runtime breakdown of the proposed glass surface depth estimation method.

Module	Runtime (ms/Frame)
Window-frame processing	7.28
RANSAC filtering and merging	58.21
Plane refitting and glass filling	11.94
Sum of listed components	77.43

## Data Availability

The source code of GlassPlane-SLAM and the download link for the UT-SRL Glass RGB-D Dataset will be made publicly available upon publication at https://github.com/Akira151/GlassPlane-SLAM (accessed on 31 October 2026).
